# Evolution and functional divergence of MADS-box genes in *Pyrus*

**DOI:** 10.1038/s41598-018-37897-6

**Published:** 2019-02-04

**Authors:** Dandan Meng, Yunpeng Cao, Tianzhe Chen, Muhammad Abdullah, Qing Jin, Honghong Fan, Yi Lin, Yongping Cai

**Affiliations:** College of Life Sciences, Anhui Agricultural University, Hefei, 230036 China

## Abstract

MADS-box transcription factors widely regulate all aspects of plant growth including development and reproduction. Although the MADS-box gene family genes have been extensively characterized in many plants, they have not been studied in closely related species. In this study, 73 and 74 MADS-box genes were identified in European pear (*Pyrus communis*) and Chinese pear (*Pyrus bretschneideri*), respectively. Based on the phylogenetic relationship, these genes could be clustered into five groups (Mα, Mβ, Mr, MIKC^C^, MIKC*) and the MIKC^C^ group was further categorized into 10 subfamilies. The distribution of MADS-box genes on each chromosome was significantly nonrandom. Thirty-seven orthologs, twenty-five *PcpMADS* (*P*. *communis* MADS-box) paralogs and nineteen *PbrMADS* (*P*. *bretschneideri* MADS-box) paralogs were predicted. Among these paralogous genes, two pairs arose from tandem duplications (TD), nineteen from segmental duplication (SD) events and twenty-three from whole genome duplication (WGD) events, indicating SD/WGD events led to the expansion of MADS-box gene family. The MADS-box genes expression profiles in pear fruits indicated functional divergence and neo-functionalization or sub-functionalization of some orthologous genes originated from a common ancestor. This study provided a useful reference for further analysis the mechanisms of species differentiation and biodiversity formation among closely related species.

## Introduction

MADS-box transcription factors have been reported in animals, plants and fungi, which initiated transcription of target gene by binding to the CArG-boxes domain in the cis-acting element of the target gene^1^. According to molecular evolutionary analysis, MADS-box genes have been classified into two large groups: type I (SRF) and type II (MEF2). In which, type I is divided into Mα, Mβ, Mr^2,3^, while type II is divided into MIKC^C^ type and MIKC* type^4,5^, and MIKC^C^ can be further classified into 12 subfamilies^6,7^. Remarkably, the MADS-box gene has a highly conserved MADS (M) domain with about 60 amino acid sequences in the N-terminal regions. The type II gene not only has the MADS (M) domain, but also contains an Intervening (I), a C-terminal (C), and a Keratin (K) domain^8,9^. Compared with type II, the type I structure is relatively simple, lacking the K domain, and its coding gene usually contains 1 to 2 exons^10,11^.

MADS-box genes play a significant role in the development and signal transduction of various organs, such as fruit development and maturation^12,13^. In the early 1990s, in order to explain the characteristics of plant floral organs, the researchers put forward the hypothesis of floral organ ABC model^14^. Based on the analysis of ABC model, class A genes specifically regulate the occurrence and development of calyx, class A and B genes together control the formation of petals, class B and C genes jointly determine the occurrence of stamens, while class C genes regulate the development of carpels. Surprisingly, recent reverse genetics studies have exposed that class D and E genes also play a vital role in regulating flower morphogenesis. Among them, class D genes mainly regulate the development of ovules^15–17^, and class E genes are mainly involved in regulating the formation and development of all floral organs^18–20^. These data further explained the occurrence and development of floral organs determined by the interaction or mutual regulation between different floral organ decisive genes^21^.

Up to now, the MADS-box gene family has been extensively studied in angiosperms, particularly in the model plant *Arabidopsis thaliana*^12^. In *Arabidopsis*, class A genes were represented by *APETALA1* (*AP1*) and *APETALA*2 (*AP2*)^22,23^, class B genes included *APETALA*3 (*AP3*) and *PISTILLATA* (*PI*)^24,25^, class C genes were represented by *AGAMOUS* (*AG*)^26^, class D genes contained *SEEDSTICK* (*STK*/*AGL11*), *SHATTERPROOF1* (*SHP1*/*AGL1*) and *SHP2* (*AGL5*)^27^, class E genes included *SEPALLATA1*, *2*, *3*, 4 (*SEP1*/2/3/4 and *AGL2*/4/9/3)^18,20,28–30^. Among these genes, besides *AP2*, all of the class A, B, C, D, and E homologous genes belong to MIKC^C^ type MADS-box genes^19,31–33^. These studies have shown that type II MADS-box genes were mainly related to plant floral organ development^34^. On the contrary, the function of type I MADS-box genes has seldom been reported, and limited reports have shown that type I MADS-box genes were mainly involved in the development of female gametophytes, endosperms or seeds^35–40^.

Pear has important economic value, and which is the third significant temperate fruit after grapes and apples. For the *Pyrus* species, our main concern is its fruit. The completion of whole-genome sequencing of European pear and Chinese pear provides a basis for further study of the MADS-box gene family function. In the present study, 73 and 74 MADS-box genes were identified from European pear and Chinese pear, respectively, and then analyzed their evolutionary relationships and genetic structure. The chromosome localization and microcollinearity analysis have also been investigated. Finally, both RNA-Seq and qRT-PCR analyses were used to understand the functional divergences of MADS-box transcription factors between European pear and Chinese pear during pear fruit development. This study will provide new ideas for further understanding the similarities and differences between closely related species at the genomic level, and for discovering the mechanisms of species differentiation and biodiversity formation.

## Results

### Identification and phylogenetic analysis of MADS-box genes

**Figure 1:**
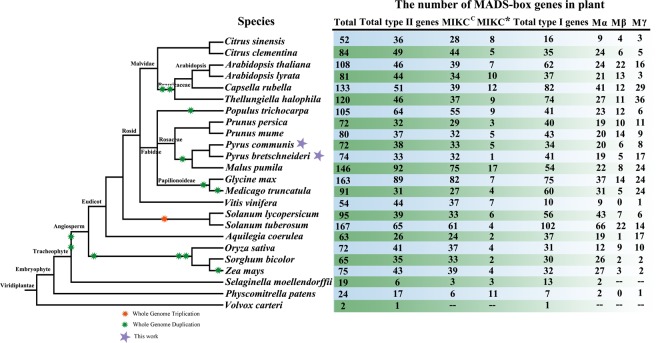
Species tree of 24 plant species and MADS-box gene numbers of each species.

**Figure 2:**
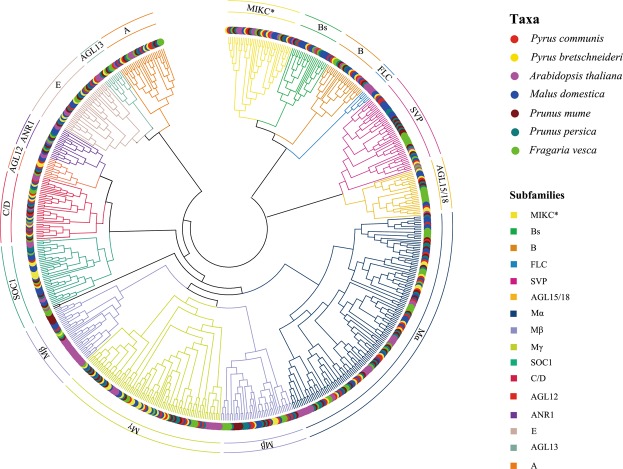
The phylogenetic tree of *Arabidopsis*, *Prunus mume*, *Prunus persica*, *Malus domestica*, *Fragaria vesca*, *Pyrus communis and Pyrus bretschneideri* MADS-box proteins was constructed using the ML method of FastTree software. The MADS-box gene of *Physcomitrella patens* (*PPMADS*) was used as the outgroup. All of the MADS-box proteins of European pear and Chinese pear can be clustered with their *Arabidopsis* counterparts except for *AtAGL47, 49, 50, 64, 82, 87, and 102*.

To identify members of the MADS-box gene family, HMM searches and BlastP were performed on the pear genome database using HMMER3 software^41^. A total of 86 PcpMADSs (*P*. *communis* MADS-box) and 95 PbrMADSs (*P*. *bretschneideri* MADS-box) candidate proteins were identified, respectively. Subsequently, the Pfam database and the SMART database were used to determine candidate MADS-box proteins containing the complete MADS domain. Ultimately, 73 and 74 members of the MADS-box gene family were identified in European pear and Chinese pear, respectively. The total number of MADS-box genes in the two *Pyrus* species was basically similar. According to the chromosomal location, these genes were named as *PcpMADS01* to *PcpMADS73* and *PbrMADS01* to *PbrMADS74*, respectively. In order to further understand the differences in the MADS-box gene family between the two *Pyrus* species, we compared the differences in exon-intron structure between European pear and Chinese pear. As shown in Table S1, we found that most orthologous genes have similar gene structure, such as *PcpMADS36*/*PbrMADS33* and *PcpMADS14*/*PbrMADS13* containing the same number of introns. To study the evolution of MADS-box gene family, a comparative genomic analysis of MADS-box genes in 24 plant species was carried out^42,43^. While most of these MADS-box gene families have been reported, the comparison of MADS-box genes in two *Pyrus* species was performed for the first time in this study. As shown in Fig. 1, the two *Pyrus* species contained a relatively large MADS-box gene family compared with *Alga*, *Bryophyta* and *Pteridophyta*, which might be caused by several WGD events. In order to better understand the evolutionary relationship of the MADS-box gene family in Rosaceae species, phylogenetic trees of MADS-box genes from *Arabidopsis*, *Prunus mume*, *Prunus persica*, *Malus domestica*, *Fragaria vesca*, *Pyrus communis and Pyrus bretschneideri* were constructed using ML and MP method, respectively. The tree topologies produced by the two algorithms were largely consistent with only minor discrepancies. Therefore, only the ML phylogenetic tree was used to further analysis in our study, and the results were basically consistent with previously studies^5,10,44,45^. In these phylogenetic trees, each of the seven species contributed at least one MADS-box gene to most subfamilies, while the *FLC* and *ANR1* subfamilies lacked members of the European pear and Chinese pear MADS-box genes. These results indicated that the MADS-box gene exhibited a conservative evolutionary relationship in the Rosaceae genomes. Subsequently, for these 73 *PcpMADS* genes and 74 *PbrMADS* genes, 40 members and 40 members could been clearly divided into type II, however, the remaining 67 members were clustered into type I based on their relationship with *Arabidopsis* MADS-box gene family^10^. Moreover, type I was further divided into three subfamilies: Mα, Mβ, Mγ, each of which had 18, 7 and 8 members in European pear, respectively, and contained 12, 5 and 17 members in Chinese pear, respectively. Remarkably, some MADS-box genes homologous to *Arabidopsis* were identified, such as, *PcpMADS21* and *AtSVP* were orthologous genes, *PbrMADS63* and *AtAP1* were orthologous genes. The number of homologous genes in *C*/*D* subfamily of European pear and Chinese pear was the same. Obviously, the number of European pear and Chinese pear type II MADS-box genes was similar in each of their subfamilies (Fig. 2, S1 and S2).

### Structural analysis of MADS-box genes

**Figure 3:**
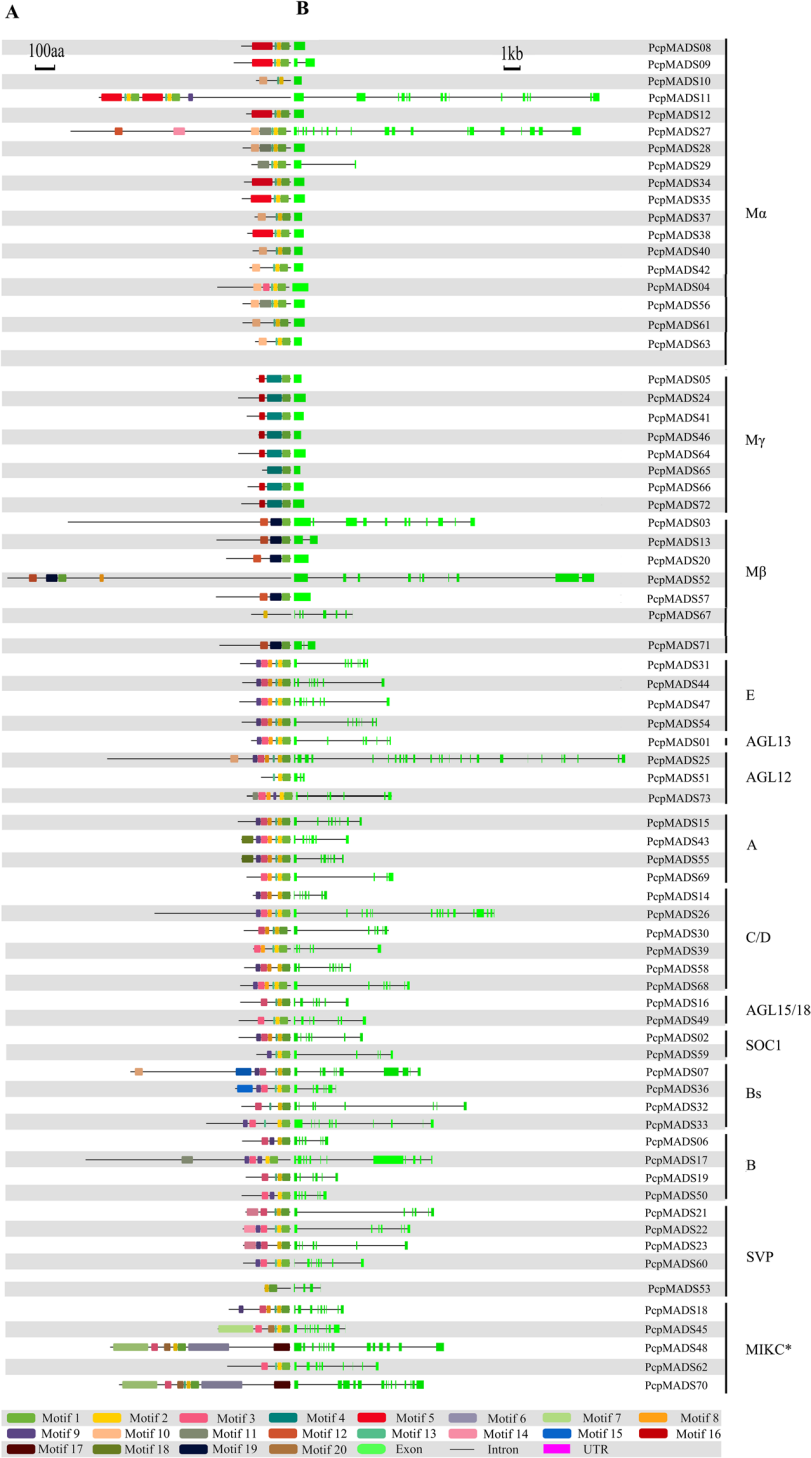
Conserved motif compositions (**A**) and the exon-intron structure (**B**) of MADS-box genes from European pear. (**A**) Twenty putative conserved motifs were elucidated using MEME with complete protein sequences. All motifs have been labeled by different colors. Details of motif were listed in Table S2. (**B**) Exons are indicated by green boxes, introns are indicated by black lines, untranslated regions (UTRs) are represented by purple boxes. The size of the exons and introns can be calculated using the top scale.

In order to understand their functional regions, a total of 147 MADS-box proteins from two *Pyrus* species were subjected to the MEME online tool^46^, to identify conserved motifs shared among orthologous proteins. In the present study, the conserved motif encoding the MADS domain was identified in each MADS-box gene of European pear and Chinese pear. Noteworthy, a total of 70 MADS-box proteins were identified as MIKC^C^-type genes in the European pear and Chinese pear genomes. However, we found that only 61 MIKC^C^-type MADS-box genes, containing the K domain and motif 1 (encoding the MADS domain), a similar motif distribution was shared by the same subfamily of PcpMADS and PbrMADS proteins, suggesting that these proteins might have similar functions. While motif 15 was only identified in the *Bs* subgroup of European pear and motif 17 was only identified in the *SOC1* subgroup of Chinese pear, these motifs might play an important role in the unique MADS-box protein (Fig. 3, S3, Tables S1 and S2). We hypothesized that these specific motifs might be important for the functional divergence of MADS-box genes.

As we all know, the function and structure of genes are closely related. To further understand the functional diversity of the MADS-box orthologous genes of two *Pyrus* species, the exon-intron structure of European pear and Chinese pear MADS-box genes was analyzed. Based on the investigation of intron numbers, we discovered that there was a significant difference in the gene structure of the type I and type II MADS-box genes. To sum up, the number of variable introns was found in type II MADS-box genes, such as most MIKC^C^-type genes contained 2 to 11 introns, and MIKC*-type genes have 8 to 16 introns. However, the type I gene structure was basically no intron (Fig. 3, S3 and Table S1). A similar phenomenon was also found in *Arabidopsis* and *Oryza sativa*^10,11^.

### Chromosomal location and duplication analysis of MADS-box genes

**Figure 4:**
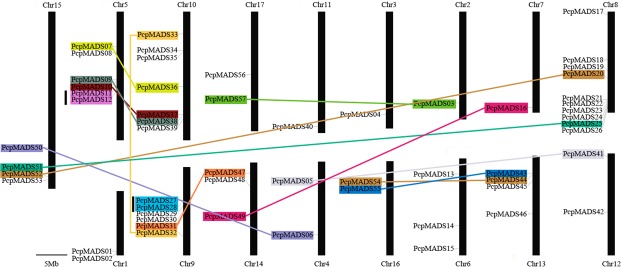
Chromosomal locations of European pear MADS-box genes. The scale refers to a 5 Mb chromosomal distance. The chromosome number is indicated at the top of each chromosome. Segmental duplication gene pairs were connected with color lines. The black vertical lines on the left of the gene names indicate tandemly duplicated genes.

According to the genome annotation file, we depicted the physical locations of the MADS-box gene on the chromosomes of European pear (57) and Chinese pear (65), respectively. The remaining 16 and 9 genes were localized on the unanchored scaffolds of European pear and Chinese pear, respectively. In European pear, MADS-box genes were distributed across all chromosomes. Chromosome 8 contained the most genes (ten), followed by chromosome 10 (seven), and five genes were distributed on five out of 17 chromosomes (chr 1, 4, 12, 16, and 17). The remaining genes were located on chromosomes 2, 3, 5, 6, 7, 9, 11, 13, 14 and 15, respectively. Furthermore, only the MIKC^C^-type gene was distributed on chromosome 1. In Chinese pear, chromosome 6 and 8 had the largest number of genes (eight) followed by chromosome 2, 13, 14 and 15 (five), and two genes were found on five out of 16 chromosomes (chr 1, 3, 5, 7 and 9). The remaining genes were located on chromosomes 4, 10, 12, 16 and 17, respectively. Significantly, there were only MIKC^C^-type genes distributed on chromosome 7. Remarkably, the MADS-box gene was distributed on each chromosome of European pear and Chinese pear, except for the Chinese pear chromosome 11. This phenomenon suggested that the MADS-box gene was widely distributed in these two genomes, which might indicated the distribution characteristics of the MADS-box gene family of Rosaceae species. Nevertheless, the distribution of the MADS-box genes on each chromosome was significantly nonrandom in both European pear and Chinese pear. In fact, a relatively high density MADS-box genes appeared on some chromosomes, for example, the European pear chromosome 8 and the Chinese pear chromosome 8, 6 (Fig. 4 and S4).

**Figure 5:**
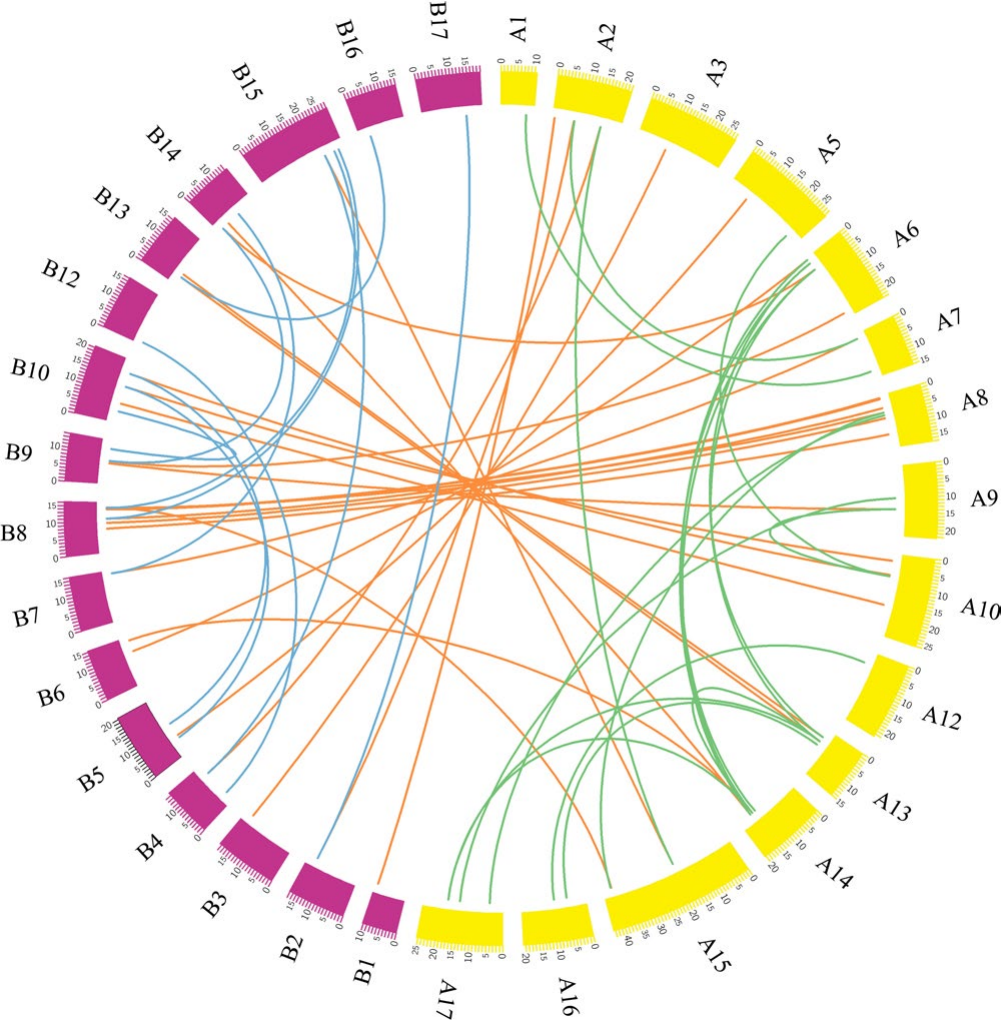
Synteny analysis of the MADS-box gene family between European pear and Chinese pear. Chinese pear chromosomes are labeled A, and are indicated by yellow boxes. European pear chromosomes are labeled B, and are represented by rose red boxes. The collinear relationship between European pear and Chinese pear is indicated by an orange line; the collinear relationship in European pear is represented by a blue line; the collinear relationship in Chinese pear is indicated by a green line.

Gene duplication was considered to be the primary source and driving force for biological evolution^47^. In this study, we performed a synteny analysis to investigate the duplication mechanism of MADS-box genes in these two *Pyrus* species. Thirty-seven orthologous gene pairs were found between European pear and Chinese pear. Twenty-five and nineteen paralogous gene pairs were detected in European pear and Chinese pear, respectively (Fig. 5, Tables S3 and S4). In the pear genome, two WGD events were discovered during the long evolutionary period, which containing an ancient WGD was estimated at ~140 MYA (i.e. Ks~1.5–1.8)^48^, and a recent WGD was estimated at 30~45 MYA (i.e. Ks~0.15–0.3)^49^. Therefore, these events might play an important role in the evolution of the pear MADS-box gene family. To investigate this possibility, we searched for the genetic similarity of the MADS-box flanking sequences (i.e. syntenic blocks) and calculated their Ks values. Moreover, we discarded any Ks values > 2.0 due to the risk of saturation^50,51^. In European pear, 12 and 11 paralogous gene pairs might be derived from the recent WGD and SD events, respectively. In Chinese pear, 8 and 3 paralogous gene pairs might be derived from recent WGD and ancient WGD events, respectively, as well as 8 paralogous gene pairs might be derived from SD events (Tables S5, S6 and S7). A MADS-box gene cluster was defined as a genomic region which containing two or more neighboring MADS-box genes and less than 200 kb, as previously reported by Holub’s (2001)^52^. Two duplicate gene pairs *PcpMADS11*/*-12*, *PcpMADS27/-28* were identified as tandem duplication (TD) events on chromosome 5 and chromosome 9 of European pear, respectively. However, no obvious gene clusters were found on each chromosome of the two *Pyrus* species (Fig. 4 and S4). These results suggested that the recent WGD or SD events have contributed to the expansion of the MADS-box gene family members in both European pear and Chinese pear. We believe that SD/WGD events must help to the acquirement of novel and unique functions related to ancestor through sub-functionalization or neo-functionalization.

### Environmental selection pressure analysis of MADS-box genes

The Ka/Ks ratios of MADS-box paralogs in European pear and Chinese pear were estimated to better understand the selection pressure acting on this gene family. In general, a Ka/Ks value less than 1 meant that these genes have experienced purifying selection with functional constraint, a Ka/Ks value of 1 indicating that these genes were neutral selection, and a Ka/Ks value more than 1 suggested these genes were under positive selection with accelerated evolution. In European pear and Chinese pear, we found that all paralogous gene pairs were under purifying selection (Ka/Ks < 1), except for *PbrMADS28* and *-62*, *PbrMADS64* and *-65*. Among them, *PcpMADS24* and *-64, PbrMADS44* and *−51* might evolve under strong purifying selection (Ka/Ks < 0.3), so we concluded that the MADS-box gene family was slowly evolving at the protein level. Additionally, some special phenomena have also been discovered, such as *PbrMADS28* and *-62* (Ka ≠ 0; Ks = 0), *PbrMADS64* and *-65* (Ka = Ks = 0) (Tables S3 and S4), which meant that they might also evolve under positive selection and strong purifying selection, respectively, because they have also been subjected to strong constraints.

**Figure 6:**
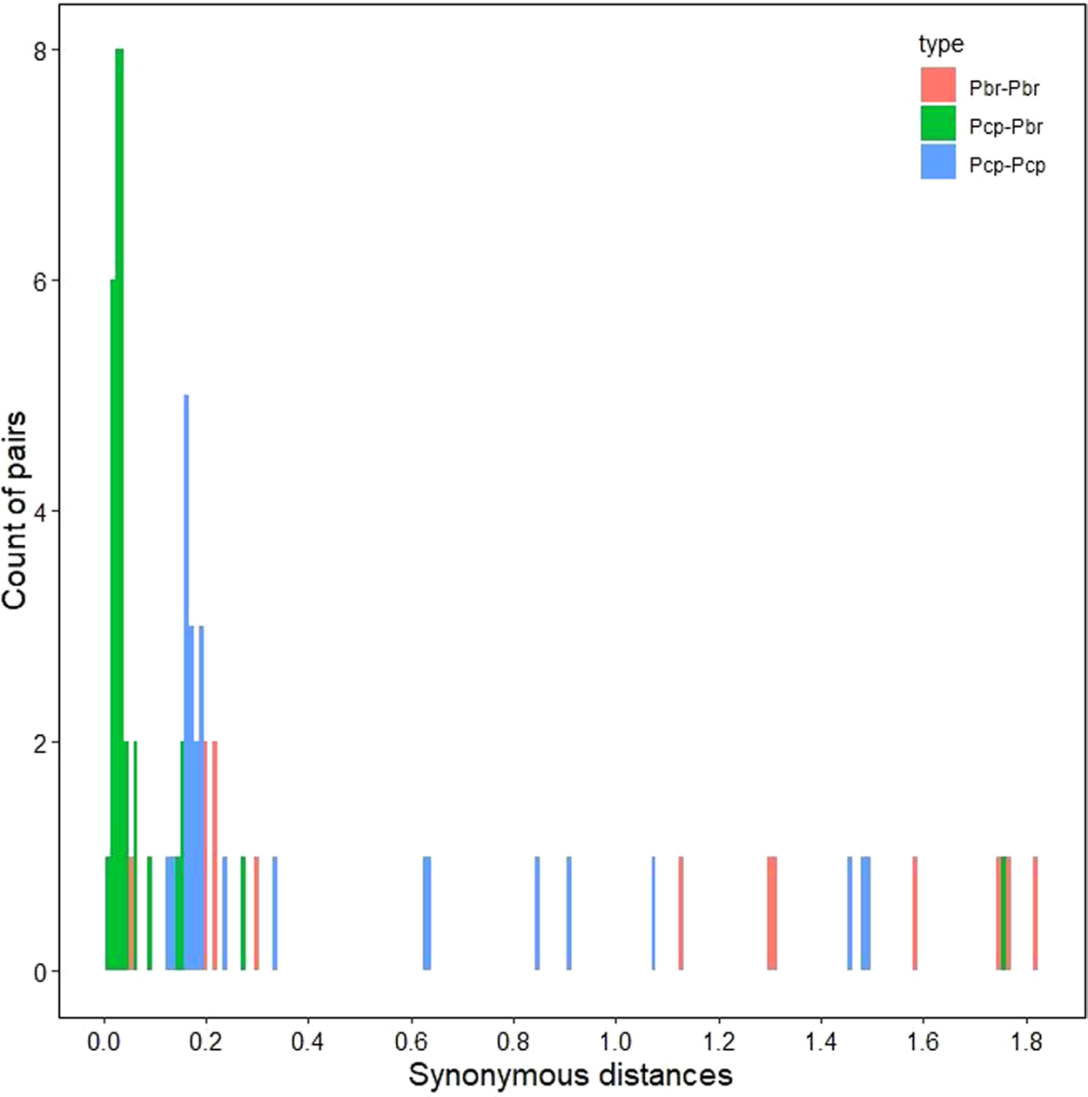
Distribution of synonymous distances (Ks) of homologous genes flanking duplicated MADS-box genes in European pear and Chinese pear. The histogram shows the number of duplicate gene pairs (y-axis) versus synonymous distance between pairs (x-axis).

Overall strong purifying selection might have masked a few individual codon sites. Therefore, a sliding-window analysis of the Ka/Ks ratios was performed for each pair of MADS-box paralogs. We found that numerous sites/regions were subjected to neutral or strong purifying selection by sliding-window analysis, which was consistent with the prediction results of the basic Ka/Ks analysis. Furthermore, we found that the majority of Ka/Ks ratios were far less than one across the coding regions, while one or a few Ka/Ks ratios were more than one (Fig. 6, S5, S6, S7, S8, S9, S10, S11 and S12). These data suggested that purifying selection was the primary driving force for the evolution of the MADS-box gene family in European pear and Chinese pear, except for one or a few coding sites.

### Functional classification analysis of MADS-box genes

**Figure 7:**
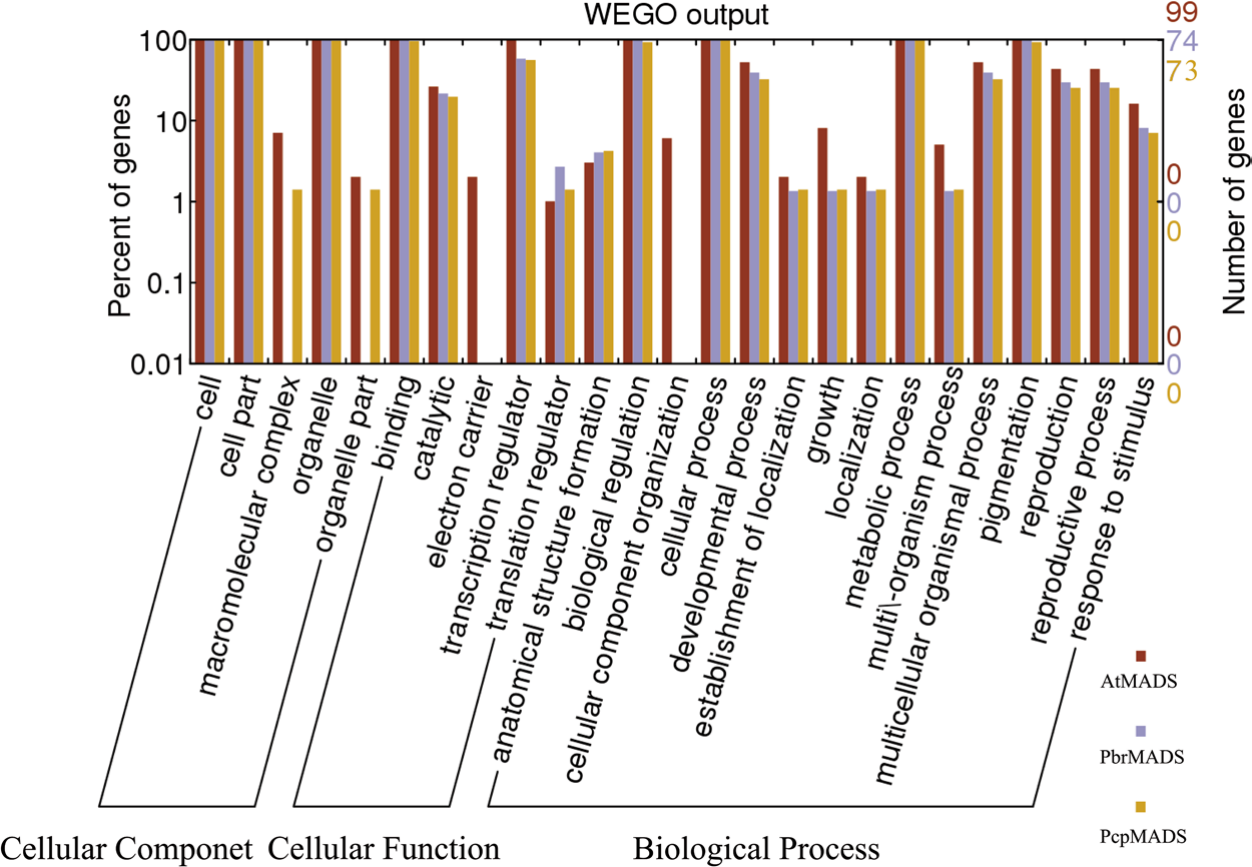
Gene Ontology (GO) analysis of MADS-box genes in *Arabidopsis*, Chinese pear and European pear.

To gain insight into the potential functions of MADS-box genes in European pear and Chinese pear, 73 and 74 MADS-box proteins were subjected to the gene ontology (GO) categories using Blast2GO software, respectively. All MADS-box genes were annotated into these three categories: cellular component, biological process and molecular function. According to the biological process, the 147 genes of European pear and Chinese pear were finally divided into 14 categories, of which the four GO terms (cellular process, pigmentation, biological regulation and metabolic process) were most over-represented. According to the molecular function, these MADS-box genes were eventually classified into 5 categories, of which the two GO terms (transcription regulator and binding) were most over-represented. Additionally, we found that these MADS-box genes were associated with cytoplasm and nucleus, and the major term was the nucleus of two *Pyrus* species based on cellular component categorization analysis (Fig. 7 and Table S8).

At present, *Arabidopsis* has the most extensive annotative evaluation of MADS-box genes in plants. In this study, we identified specific subfamilies that help to characterize each subgroup of the pear MADS-box gene family (Table S9). For instance, subgroup *AGL15/*18 included two *PcpMADS* genes, four *PbrMADS* genes and two *AtMADS* genes (*AtAGL15* and *AtAGL*18), which play an important role in repressing the floral transition in *Arabidopsis*. Therefore, we hypothesized that the *PcpMADS* and *PbrMADS* genes in this subfamily might also play crucial roles in the floral repression pathway of pear. Similarly, in subgroup *A*, four *AtMADS* genes regulated the occurrence and development of sepal and peta. Thus, we predicted that the five *PcpMADS* genes and the three *PbrMADS* genes in this subgroup might also have similar functions. Anthocyanin is one of the key factors affecting the quality of pear fruit. Therefore, we also explored the role of the pear MADS-box gene in the flavonoid metabolic pathway. Using RNA-seq data^53^, we identified 21 MADS-box genes with divergent expression. Among them, the *PbrMADS20*, *PbrMADS45*, *PbrMADS46* and *PbrMADS58* genes were orthologs of *MdMADS8* and *MdMADS9*. Previous studies have confirmed that these two *MdMADS* genes can regulate anthocyanin biosynthesis. In our study, the *PbrMADS20*, *PbrMADS45*, *PbrMADS46* and *PbrMADS58* genes were identified as divergently expressed genes between red/green skin color mutants of pear, so we speculated that these four *PbrMADS* genes might be the key factors regulating the anthocyanin biosynthesis of pear (Fig. S13).

### Expression correlation of orthologous of MADS-box genes

**Figure 8:**
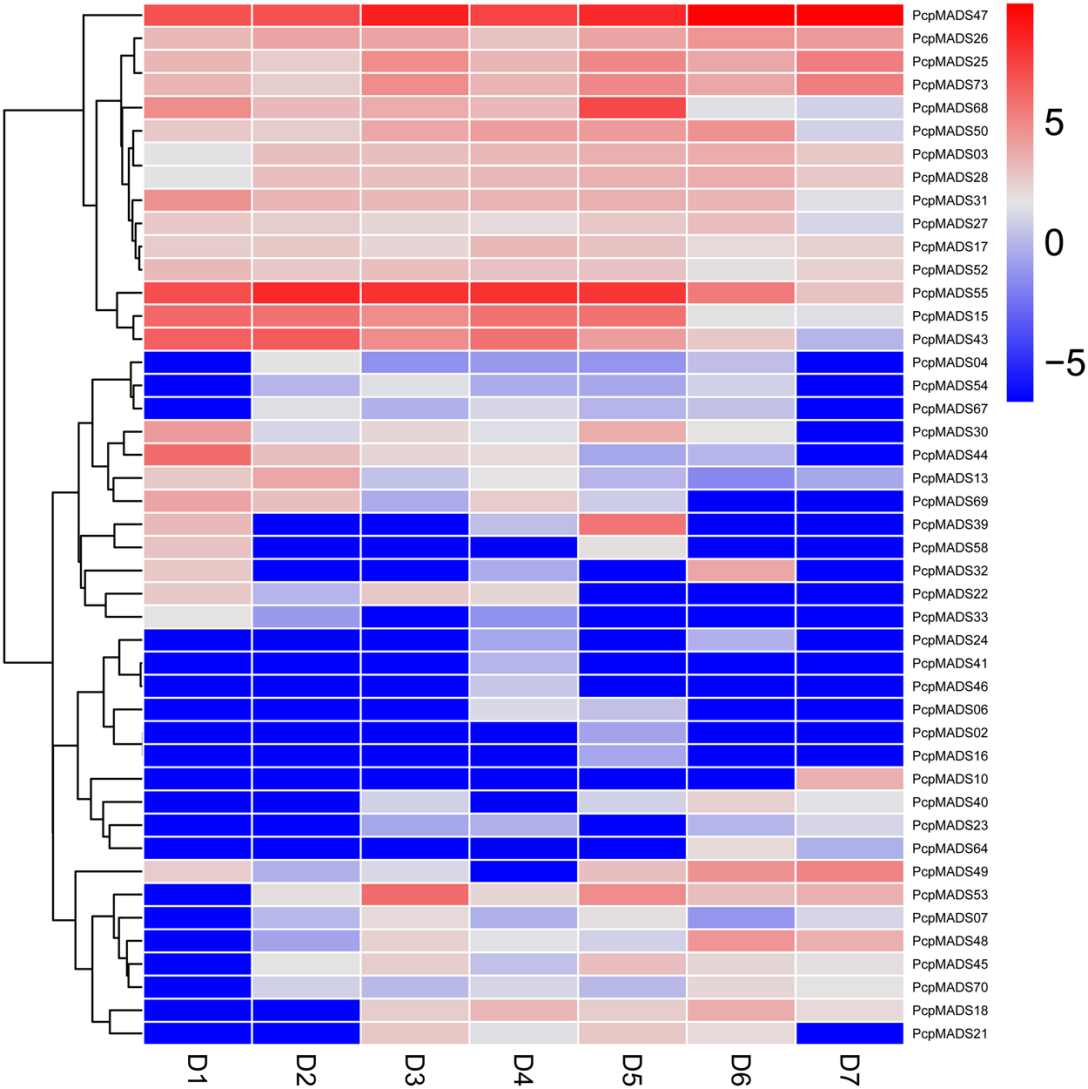
Expression profiling of European pear MADS-box genes during seven developmental stages of pear fruit. D 1 to D 5 indicated 15, 30, 55, 85 and 115 days after full blooming, respectively. D 6 indicated mature stage. D 7 indicated fruit senescence stage. The expression profile data was obtained by RNA-Seq data. Red and blue colors indicate high-expression and low-expression, respectively.

Gene expression patterns can provide important clues for studying gene function. In order to further understand the function and divergence of the MADS-box gene during pear fruit development, the expression patterns of MADS-box genes in European pear and Chinese pear were compared using transcriptome sequencing data. These data shown that 45 (61.6%) and 24 (32.4%) members of the MADS-box gene family were expressed in European pear and Chinese pear, respectively (Fig. 8 and S14). Usually, orthologous genes are considered to exhibit similar expression patterns. We used the Pearson’s correlation coefficient (r) method to calculate the expression correlation between orthologous MADS-box genes in pear to test their expression diversity^54,55^. Finally, nine orthologous gene pairs were found to be non-divergent, such as *PcpMADS58*/*PbrMADS50*, *PcpMADS70*/*PbrMADS14* (r > 0.5). Five orthologous gene pairs were divergent, for example, *PcpMADS26*/*PbrMADS56*, *PcpMADS50*/*PbrMADS53*, *PcpMADS45*/*PbrMADS43* (r < 0.3). However, only two orthologous MADS-box gene pairs were ongoing divergent (*PcpMADS31*/*PbrMADS20* and *PcpMADS06*/*PbrMADS07*) (0.3 < r < 0.5) (Table S4).

### Expression profile analysis of MADS-box genes

**Figure 9:**
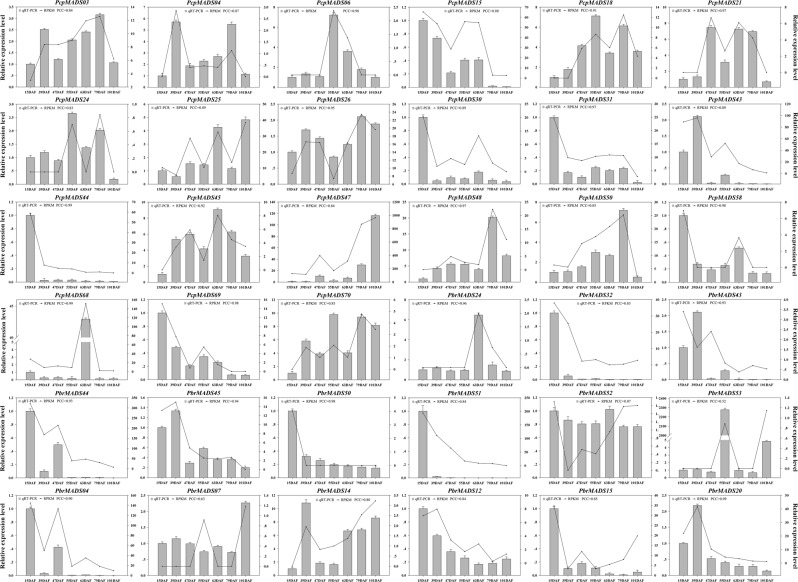
Expression patterns of European pear and Chinese pear MADS-box genes during six developmental stages of pear fruit at 15, 39, 47, 55, 63 and 79 days after flowering (DAF). These expression profile data were obtained using qRT-PCR.

By analyzing the gene expression profiles of different fruit development stages in the two *Pyrus* species, the potential role of MADS-box genes during pear fruit development was revealed. As shown in Fig. 8 and S14, in general, members from the same subfamily presented similar expression patterns with minor differences in expression levels. In European pear and Chinese pear, except for *PcpMADS54*, *E* subfamily members were highly expressed in D1 (15 days after full blooming) than in other stages, and expression levels decreased during fruit development, indicating that they might contribute to the early development of the fruit. However, some genes from different subfamilies exhibited similar expression patterns, such as, the expression levels of *PcpMADS10* and *PbrMADS49* were higher in D7 (fruit senescence stage) than in other stages, and the expression levels increased during fruit development, suggesting that they might contribute to the late stages of fruit development. Moreover, *PcpMADS13*, *PbrMADS12* and *PbrMADS32* were highly expressed in D2 (30 days after full blooming), although the expression of *PcpMADS13*, *PbrMADS12* and *PbrMADS32* was diverse in different stages of fruit development, this phenomenon indicated that these three MADS-box genes had common roles in different stages of fruit development. Remarkably, we found that some MADS-box genes were expressed at all developmental stages, such as *PcpMADS47*, *PcpMADS55*, *PbrMADS04*, *PbrMADS44* and *PbrMADS46*, indicating these genes might play regulatory roles at multiple developmental stages. Additionally, we found that 28 *PcpMADS* genes and 50 *PbrMADS* genes lacked expression information at all stages of pear fruit development, implying these genes were expressed in other organs, such as stem, root, leaf, flower, or under special conditions. Interestingly, we found most orthologous gene pairs contained similar expression patterns in both European pear and Chinese pear, such as, *PcpMADS36*/*PbrMADS33* and *PcpMADS08*/*PbrMADS11* had no transcriptional signal, *PcpMADS58*/*PbrMADS50* were highly expressed in D1 (15 days after full blooming), *PcpMADS43*/*PbrMADS44* and *PcpMADS44*/*PbrMADS45* were detected as down-regulated during fruit development. In addition, the expression of some orthologous genes had functional divergence. Such as, *PcpMADS47* was highly expressed in D7 (fruit senescence stage), while its orthologous gene *PbrMADS15* was expressed at a high level in D1 (15 days after full blooming); *PcpMADS26* was expressed at all stages of fruit development, and its orthologous gene *PbrMADS56* was essentially not expressed at all stages of fruit development. These results suggested that these orthologous genes had functional divergence when they evolved from the common *Pyrus* species. The qRT-PCR was performed to further verify the expression pattern of MADS-box genes during pear fruit development. The results suggested that the expression levels of these genes were basically consistent with that of transcriptome sequencing data (Pearson’s correlation coefficient (PCC) >0.2). For example, in our experiments, the expression of these genes with Ct values greater than 36 was not detected in transcriptome sequencing data, or their expression levels were low. Additionally, some differences were also found, such as *PbrMADS52* had a high expression value in D1 in transcriptome data, but was highly expressed in D5 (63 days after full blooming) in qRT-PCR (PCC = 0.07) (Fig. 9 and Table S10). The reason for these divergences might be the difference in sample growth.

## Discussion

Systematic identification and analysis of the MADS-box gene family has been performed in a variety of plants, such as *A*. *thaliana*^10^, rice^11^ and others. In this study, identification, evolution, classification and expression profile analysis were performed in these two *Pyrus* species (i.e. European pear and Chinese pear). A total of 73 and 74 MADS-box genes were identified in European pear and Chinese pear, respectively. However, Wang *et al*. (2017) identified 95 MADS-box genes in Chinese pear, which was higher than the number of Chinese pear MADS-box genes in our study, this might be due to the use of different methods^56^. In the present study, we used a more stringent E-value threshold of 1e^−3^, which was consistent with previous studies^44^. Moreover, these sequences comprising partial sequences or partial MADS domains have been excluded, as previously published papers have demonstrated that incomplete gene sequences were used in analyses could led to incorrect results^57,58^. Therefore, the number of MADS-box gene family members was more accurate in our study.

In addition, many of the features of MADS-box genes have been confirmed in our study, and were consistent with other species. Remarkably, we also discovered some novel characteristics of European pear and Chinese pear compared with *Arabidopsis* and *O*. *sativa*^10,11^. In our study, the type II MADS-box genes from European pear and Chinese pear could be further clustered into 11 subfamilies, based on the phylogenetic relationship with *Arabidopsis* type II genes. Interestingly, we have not identified the MADS-box gene of the *ANR1* subfamily from European pear and Chinese pear. Additionally, the *FLC* subfamily has been demonstrated to be able to regulate flowering time through autonomous and vernalization pathways^59^. In the current study, the *FLC* subfamily was not found in European pear and Chinese pear, this phenomenon has also been reported in the *P*. *mume* and *M*. *domestica* genomes^44,45^. These results indicating other pathways might play roles in floral transition, such as photoperiod or gibberellic acid (GA)-dependent pathway.

In our study, the analysis of several conserved exon-intron structures and motifs within the conserved MADS domain existed not only in European pear and Chinese pear, but also in primitive plants, such as *Physcomitrella patens*, *Volvox carteri* and *Medicago truncatula*, indicating their key roles in plant developmental processes and ancient origin during the long evolutionary period. Indeed, this hypothesis could be supported by several published papers: (1) Flowering time and floral organ development: a growing number of key MADS-box genes including *FLC*, *SOC1*, *SVP*, *AGL24*, *AGL16*, *AGL15* and *AGL18*, as well as ABCDE model genes were involved in flowering and floral organogenesis^60,61^. (2) Fruit ripening: Vrebalov *et al*.^62^ found that a MADS-box gene in tomato was closely related to fruit maturation^62^. (3) Ovule development: Xu *et al*. (2002) demonstrated that *HoMADS1* played an important role in determining the characteristics of carpels and ovules in hyacinth flowers^63^. (4) Vegetation growth: Tapia-Lopez *et al*.^64^ discovered that *AGLl2 (XALI)* affected the vegetative growth of plants^64^.

Gene duplication is the main evolutionary mechanism for generating novel genes, helping organisms adapt to different environments. TD, SD and WGD are well known plant gene duplication patterns^65^. In our study, we found that a high proportion of MADS-box genes were distributed in syntenic blocks, which indicated that SD/WGD events might contribute significantly to the expansion of the MADS-box gene family. Moreover, we found that the type II MADS-box gene had more SD/WGD gene pairs (26) than type I (16), which might be the cause of the type II MADS-box gene numbers more than the type I. Obviously, these data also shown that 38 out of 74 (51.3%) MADS-box genes were involved in SD/WGD events in Chinese pear, which was lower than European pear (63.0%) (Tables S5, S6 and S7). Additionally, we also identified two TD events in European pear (Fig. 4 and S4). In conclusion, SD/WGD events might play an important role in the expansion of MADS-box gene family members in the European pear and Chinese pear genomes.

In the MADS-box gene family, different subfamilies displayed different expression profiles in various species, such as strawberry, mei, rice and *Arabidopsis*^11,37,44,57^. For example, previous studies have suggested that *Arabidopsis AGL*17 was specifically expressed in roots^10^. However, members of *AGL17* in other species were expressed in other tissues, such as fruits^11,66^. In addition, the* ALG13*, *AGL15/18*, MIKC*, *A*, *B*, *C*/*D*, *E* members of the above species were primarily expressed in flowers and/or fruits^6,11^. In the present study, the expression of pear MADS-box genes at different stages of fruit development was analyzed using RNA-Seq data and qRT-PCR. Interestingly, members of the *ALG13*, *AGL15*/*18*, MIKC* and ABCDE model genes were found to be expressed primarily at different stages of the fruit, implying these genes might be involved in the regulation of fruit development. It is well known that the *FUL* gene (class A), *SHP1* and *SHP2* gene (class C) and the *STK* gene (class D) were involved in regulating the development of endosperm, central cells and seeds in *Arabidopsis*^17,67^. In strawberry, the *FaMADS9* gene belonging to class E was identified to be involved in fruit development and maturation. In tomato, members of the *C* subfamily *TAGL1* and *TAG1*, and *E* subfamily member *TM29* were identified to play a role in fruit development and ripening^68^. Additionally, *PmMADS14*, -*17* and *-28* in the *SEP* subfamily (class E), and *PmMADS27* in the *AG* subfamily (class C) also exhibited high expression levels^44^. In the present study, these genes belonging to the *C*/*D* subfamily were found to exhibit diverse transcript accumulation patterns in fruit development and maturation by RNA-Seq data and qRT-PCR analysis. According to these analyses, we believe that their diverse expression patterns indicated their special roles in different fruit development stages. Remarkably, the gene expression patterns of paralogous and/or orthologous were also investigated. We found that most of these gene pairs had similar expression patterns, indicating functional redundancy. However, several orthologous gene pairs have been found with diverse expression patterns, which help to understand the difference in fruit quality between European pear and Chinese pear.

In the present study, 147 MADS-box gene family members were identified and divided into five subgroups (MIKC^C^, MIKC*, Mα, Mβ and Mγ) based on the MADS-box gene from *Arabidopsis*. Subsequently, systematic comparative analysis was carried out, including physical locations, synteny, gene structure, and expression pattern analysis using RNA-Seq and qRT-PCR. The expression pattern of the MADS-box gene family has increased our knowledge of the genes involved in the development and maturation of European pear and Chinese pear fruit. These data will provide valuable information for further analysis the mechanisms of biodiversity formation and species differentiation among closely related species.

## Materials and Methods

### Identification and phylogenetic analysis of MADS-box genes

The European pear and Chinese pear genome sequences were downloaded from the Genome Database for Rosaceae (https://www.rosaceae.org/) and the GigaDB (http://gigadb.org/site/index), respectively. The MADS-box gene of *Arabidopsis* was obtained from previously published articles^10^. The HMM model of the MADS-box transcription factor (PF00319) was derived from the Pfam database (http://pfam.xfam.org/). Then the MADS-box candidate gene sequence was screened by HMMER3 software (E-value = 0.001)^41^ and BlastP software, respectively. These putative MADS-box sequences were confirmed to contain the complete MADS domain through the Pfam database and the SMART database for further analysis. Multiple sequence alignment of MADS-box gene sequences was performed using MUSCLE with MEGA 6.0 software^69^. The best alternative model for all MADS-box genes was determined by model testing software, and the most suitable for MADS-box genes was the JTT method of FastTree version 2.1^70^. The maximum likelihood (ML) tree was constructed by FastTree with 1000 bootstrap replicates. The Maximum Parsimony (MP) methods of the MPBoot software^71^ was also used to create a phylogenetic tree and to validate the results of the ML method.

### Conserved motif and gene structure analysis of MADS-box genes

The gene and cDNA sequences of the European pear and Chinese pear MADS-box genes were obtained from the GDR database and the GigaDB database, respectively. Their exon-intron structure was analyzed by Gene Structure Display Server (http://gsds.cbi.pku.edu.cn/index.php)^72^. Afterwards, the conserved motifs of the European pear and Chinese pear MADS-box proteins were identified by the MEME online analysis tool (http://meme-suite.org/)^46^, with the following parameters: the maximum value of motifs was set to identify 20 motifs, the minimum motif width was 6, and the maximum motif width was 200. Finally, the obtained conserved motifs were annotated by Pfam and SMART.

### Chromosomal locations and synteny analysis of MADS-box genes

European pear and Chinese pear GFF annotation files were downloaded from the Rosaceae genome database and the GigaDB database, respectively. Subsequently, the location maps of the European pear and Chinese pear MADS-box gene were drawn by MapInspect software (http://mapinspect.software.informer.com/), based on the starting position of these genes on the chromosome.

Combined with the gene identifier file, the gene list file and the coding sequence file of European pear and Chinese pear MADS-box genes, the microsynteny analysis of MADS-box paralogous and orthologous genes from European pear and Chinese pear were carried out using MCScanX^73^ (E-value ≤ 10^−5^).

In the pear genome, two WGD events were discovered during the long evolutionary period, which including a recent WGD was estimated at 30~45 MYA (i.e. Ks ~0.15–0.3)^49^, and an ancient WGD was estimated at ~140 MYA (i.e. Ks ~1.5–1.8)^48^. In this study, we defined the Ks values in these two ranges as recent WGD and ancient WGD, respectively, and the remaining Ks values might be SD or large-scale duplication events.

Fourfold synonymous third-codon transversion rates (4DTV) between duplicated gene pairs was estimated to validate the results of Ks values.

### Environmental selection pressure analysis of MADS-box genes

The nonsynonymous (Ka)/synonymous (Ks) substitution rate was a molecular evolutionary parameter for measure the selection pressure. The mean Ks values of the flanking conserved genes in syntenic blocks was calculated by DnaSP (version5.10)^74^. In addition, a sliding window analysis of the Ka/Ks ratio was carried out with the following parameters: window size, 150 bp; step size, 9 bp.

### Gene Ontology (GO) annotation analysis of MADS-box genes

The GO database mainly includes three sections: biological process, molecular function and cellular component. In our study, Blast2GO software^75^ was used to implement Gene Ontology (GO) annotation analysis. Visualization of GO classifications was performed using the WEGO online tool^76^.

### RNA isolation and qRT-PCR

In this study, we collected six fruit samples at 15 days after flowering (DAF), 39 DAF, 47 DAF, 55 DAF, 63 DAF and 79 DAF in the Dangshan Orchard of Anhui Agricultural University. Based on the manufacturer’s protocol, the RNAprep pure Plant Kit (Tiangen, Beijing) was used to extract total RNA from all samples. 1 µg of total RNA was used to synthetize the first-strand cDNA using the PrimeScript TM RT reagent Kit (Takara, Dalian). The Beacon Designer 7 software was used to design the primers of the pear MADS-box gene and the pear tubulin gene^77^, and then these primers were synthesized by General Biosystems (Chuzhou, China). qRT-PCR was performed using a Bio-rad CFX96 TouchTM Deep Well Real-Time PCR Detection System with the following cycling profile: 98 °C for 2 min, followed by 40 cycles at 98 °C for 10 s, 60 °C for 10 s and 68 °C for 30 s. The Ct value method^78^ was carried out to estimate the relative expression ratio of these *PcpMADSs* and *PbrMADSs* genes. In the current study, three biological repetitions were performed in all real-time RT-PCR experiments.

### Expression correlation of orthologous MADS-box genes

In this study, all statistical analyses were executed using the R 3.4.1 tool. The Pearson’s correlation coefficient (r) method was used to evaluate the similarity between the expression profiles of each orthologous gene pair^54,55^. According to previous studies, divergent, ongoing divergent and non-divergent were indicated by r < 0.3, 0.3 < r < 0.5 and r > 0.5, respectively^54,55^.

### Expression profile analysis

The RNA-Seq data of European pear and Chinese pear was downloaded from the NCBI SRA database (SRP070620 and SRP065003). Then the RNA-Seq data was filtered by the pipeline Fastq_clean^79^ to remove low quality reads and mapped onto the genomic sequences using the pipeline tophat2 with default parameters. The number of reads mapped was normalized by the FPKM (fragments per Kilobase million) method, and the different expressed genes were detected by Cufflinks^80^. Finally, the heatmap of MADS-box genes was exhibited using the R 3.4.1 tool.

## Supplementary information

supplementary information

## Data Availability

All data generated or analysed during this study are included in this published article (and its Supplementary Information files).
