# Supplementary material for: Evolution and functional divergence of MADS-box genes in Pyrus
Source: Sci Rep. 2019 Feb 4;9:1266. doi: 10.1038/s41598-018-37897-6 (PMC6362034; doi:10.1038/s41598-018-37897-6)
Supplement: Supplementary file 1 — supplementary information [file 41598_2018_37897_MOESM1_ESM.pdf]

Evolution and functional divergence of MADS-box genes in *Pyrus*

Dandan Meng, Yunpeng Cao, Tianzhe Chen, Muhammad Abdullah, Qing Jin, Honghong Fan, Yi Lin*, Yongping Cai*

School of Life Sciences, Anhui Agricultural University, Hefei 230036, China.

*Corresponding author: Yi Lin, linyi320722@163.com

Yongping Cai, swkx12@ahau.edu.cn

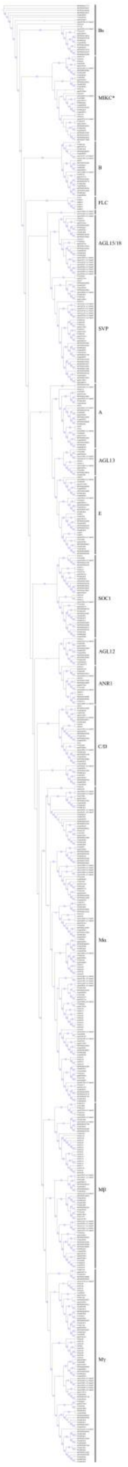

Fig.S1. The phylogenetic tree of *Arabidopsis, Prunus mume, Prunus persica, Malus domestica, Fragaria vesca, Pyrus communis and Pyrus bretschneideri* MADS-box proteins was constructed using the ML method of FastTree software. The MADS-box gene of *Physcomitrella patens* (PPMADS) was used as the outgroup. All of the MADS-box proteins of European pear and Chinese pear can be clustered with their *Arabidopsis* counterparts except for *AtAGL47, 49*, *50*, *64*, *82*, *87,* and *102*.

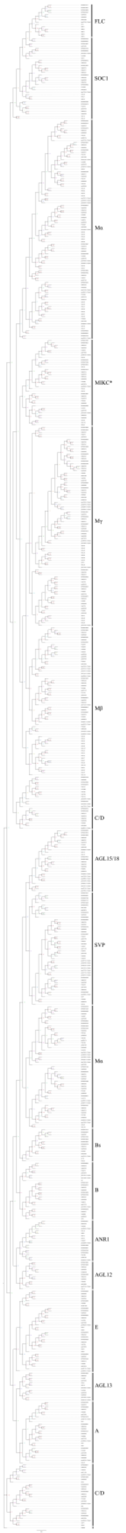

Fig.S2. The phylogenetic tree of *Arabidopsis, Prunus mume, Prunus persica, Malus domestica, Fragaria vesca, Pyrus communis and Pyrus bretschneideri* MADS-box proteins was constructed using the MP method of MPBoot software. The MADS-box gene of *Physcomitrella patens* (PPMADS) was used as the outgroup. All of the MADS-box proteins of European pear and Chinese pear can be clustered with their *Arabidopsis* counterparts except for *AtAGL47*, *49*, *50*, *64*, *82*, *87*, and *102*.

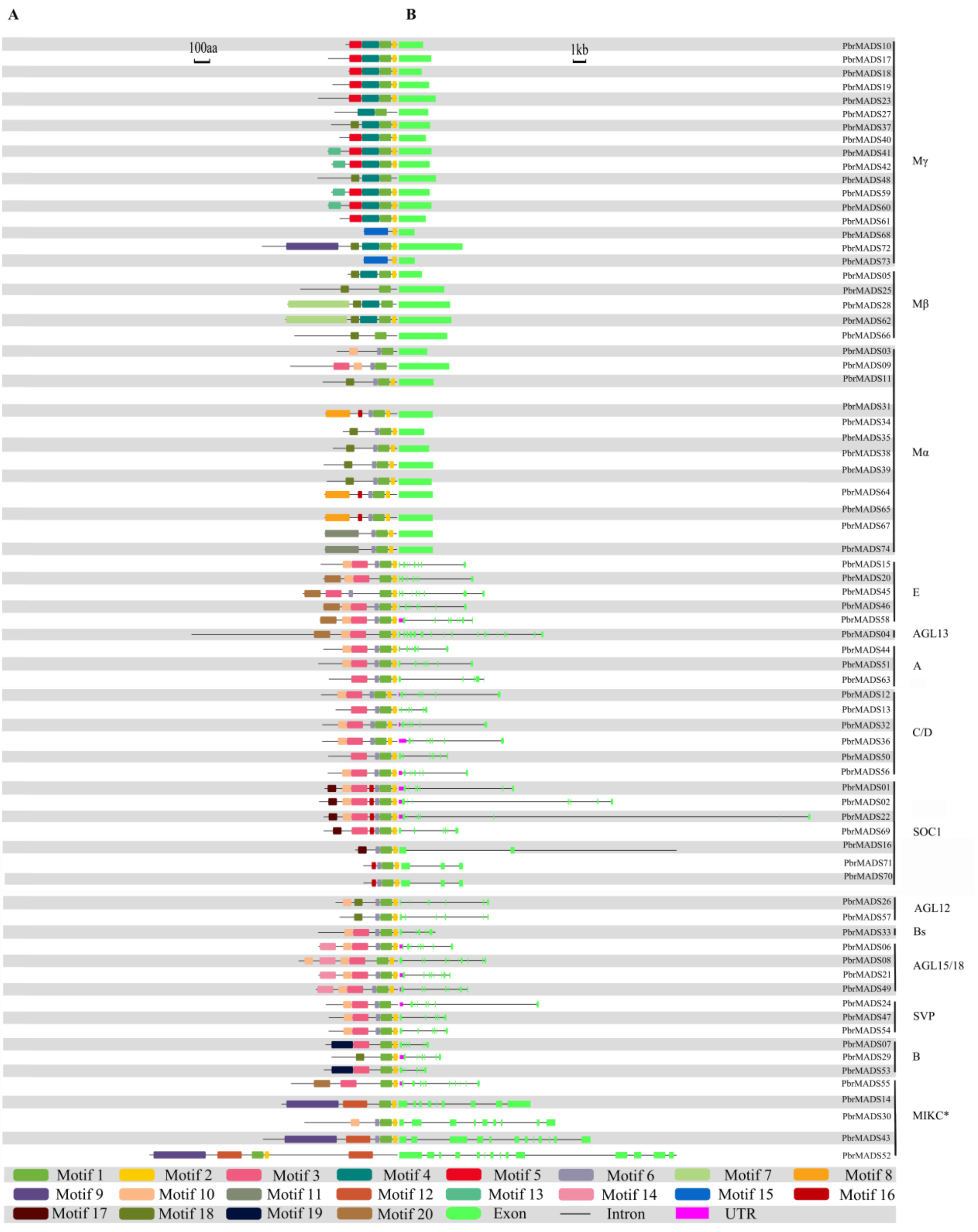

Fig.S3. Conserved motif compositions (A) and the exon-intron structure (B) analysis of MADS-box genes from Chinese pear. (A) Twenty putative conserved motifs were elucidated using MEME with complete protein sequences. All motifs have been labeled by different colors. Details of motif were listed in Table S2. (B) Exons are indicated by green boxes, Introns are represented by black lines, untranslated regions (UTRs) are indicated by purple boxes. The size of the exons and introns can be calculated using the top scale.

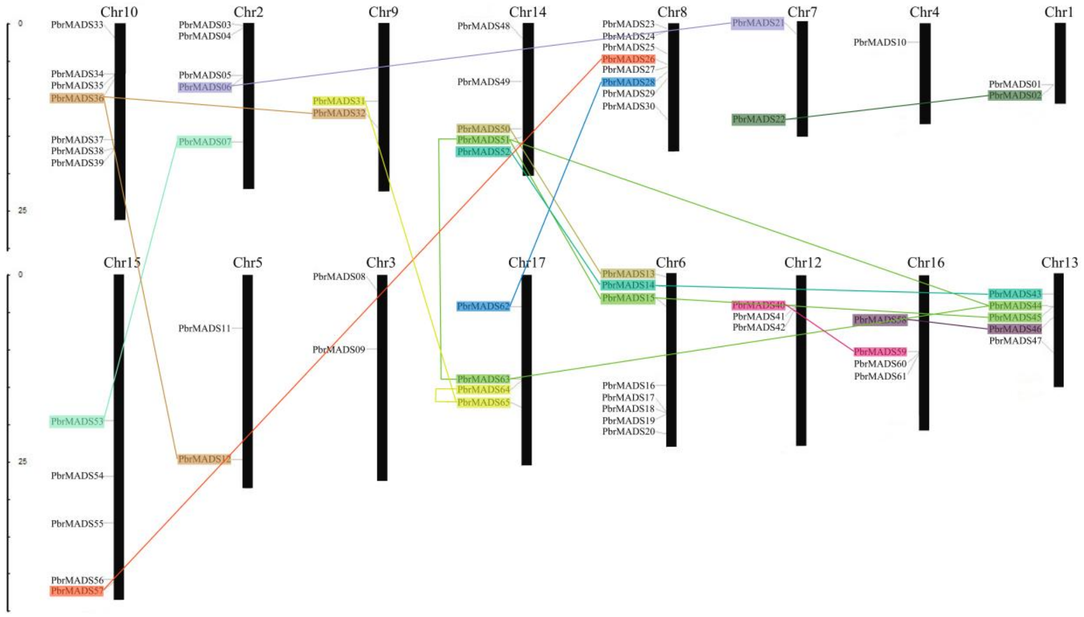

Fig.S4. Chromosomal locations of Chinese pear MADS-box genes. The chromosome number is indicated at the top of each chromosome. The scale refers to a 5 Mb chromosomal distance. Segmental duplication gene pairs were connected with color lines.

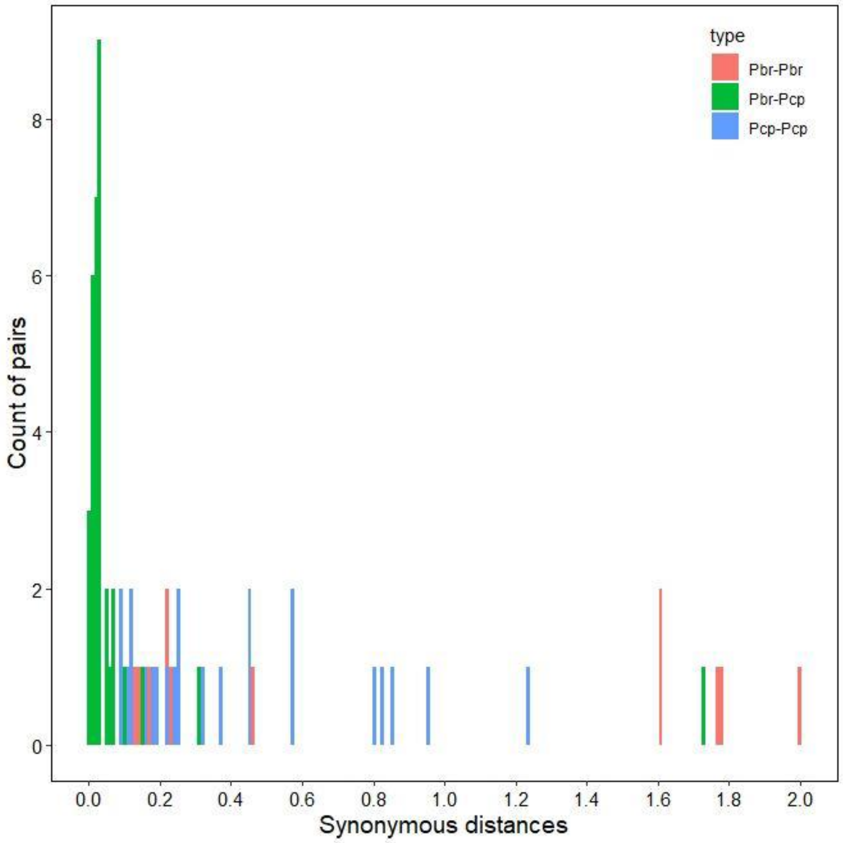

Fig.S5. Distribution of synonymous distances (Ks) of homologous MADS-box genes in European pear and Chinese pear. The histogram shows the number of duplicate gene pairs (y-axis) versus synonymous distance between pairs (x-axis).

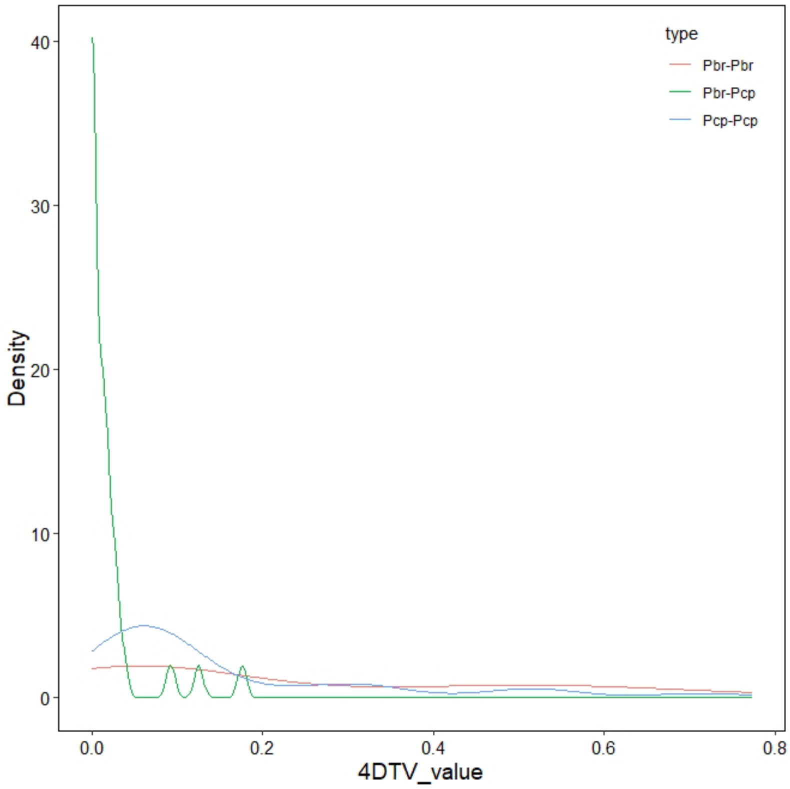

Fig.S6. 4DTV values between all predicted duplicated MADS-box genes in European pear and Chinese pear.

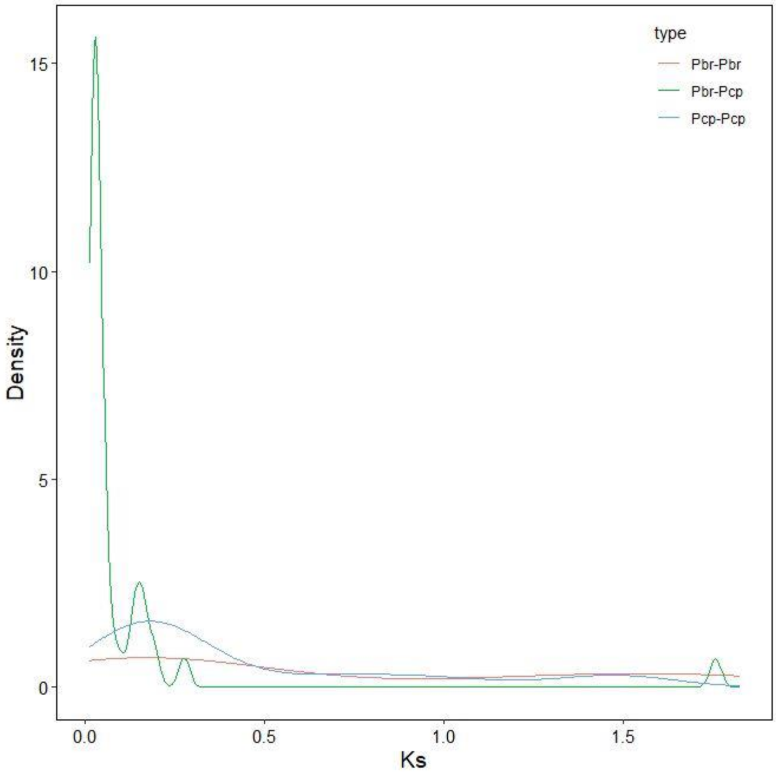

Fig.S7. Ks values between all predicted duplicated MADS-box genes in European pear and Chinese pear.

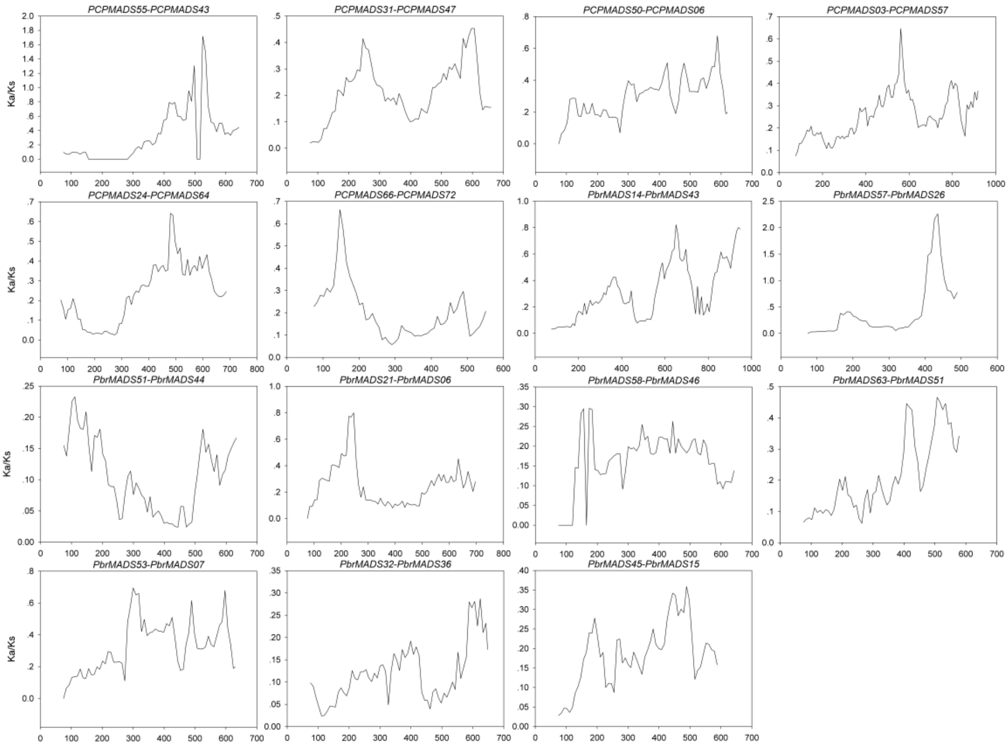

Fig.S8. Sliding-window analysis of MADS-box paralogous gene pairs with Ka/Ks values less than 0.3 in European pear and Chinese pear. The x-axis denotes the nucleotide positions within each gene. The window size was 150 bp, and the step size was 9 bp.

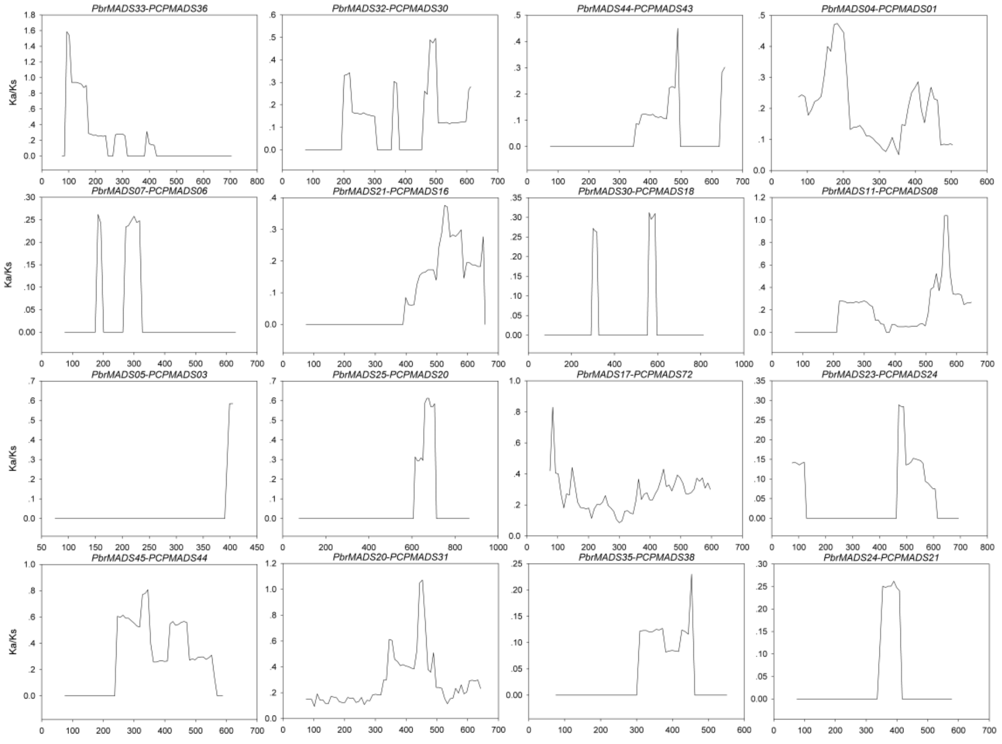

Fig.S9. Sliding-window analysis of MADS-box orthologous gene pairs with Ka/Ks values less than 0.3 between European pear and Chinese pear. The x-axis denotes the nucleotide positions within each gene. The window size was 150 bp, and the step size was 9 bp.

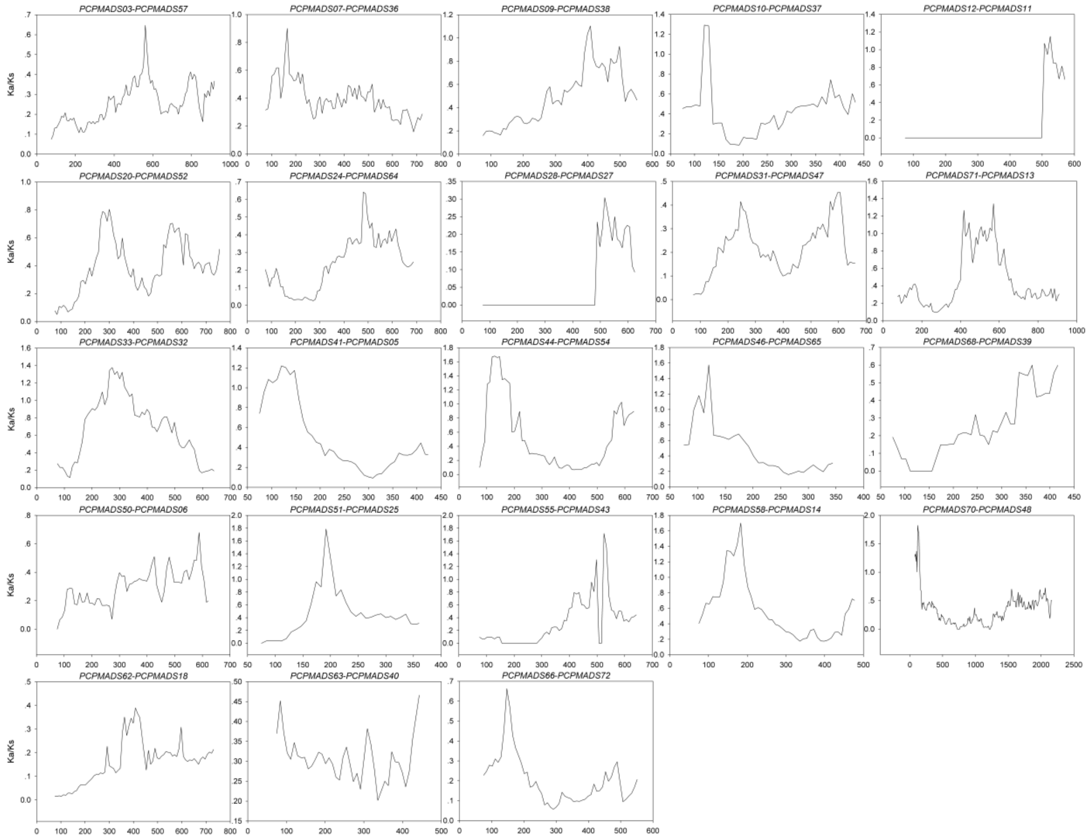

Fig.S10. Sliding-window analysis of MADS-box paralogous gene pairs in European pear. The x-axis denotes the nucleotide positions within each gene. The window size was 150 bp, and the step size was 9 bp.

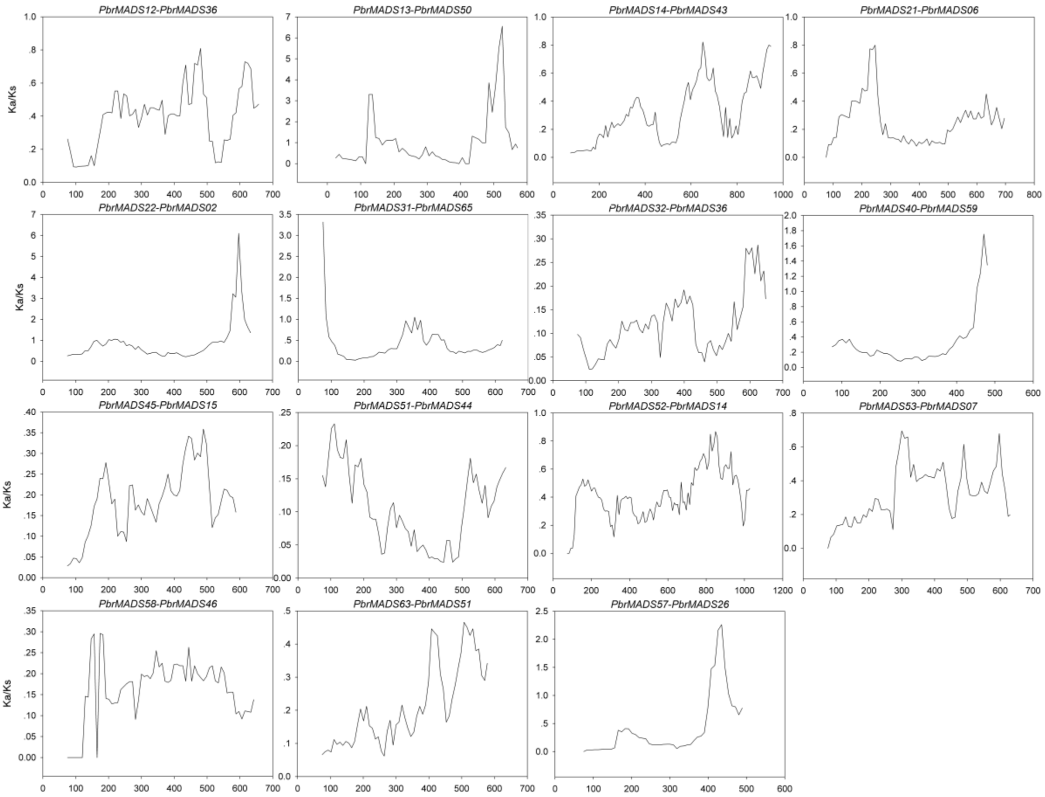

Fig.S11. Sliding-window analysis of MADS-box paralogous gene pairs in Chinese pear. The x-axis denotes the nucleotide positions within each gene. The window size was 150 bp, and the step size was 9 bp.

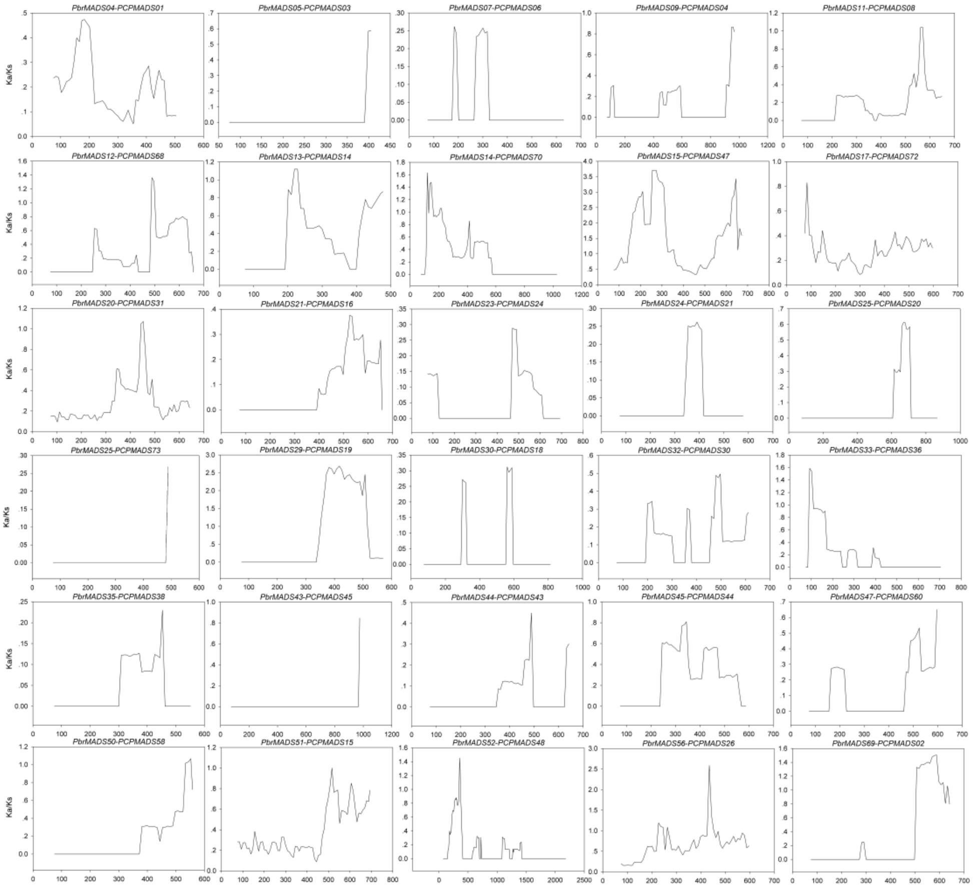

Fig.S12. Sliding-window analysis of MADS-box orthologous gene pairs between European pear and Chinese pear. The x-axis denotes the nucleotide positions within each gene. The window size was 150 bp, and the step size was 9 bp.

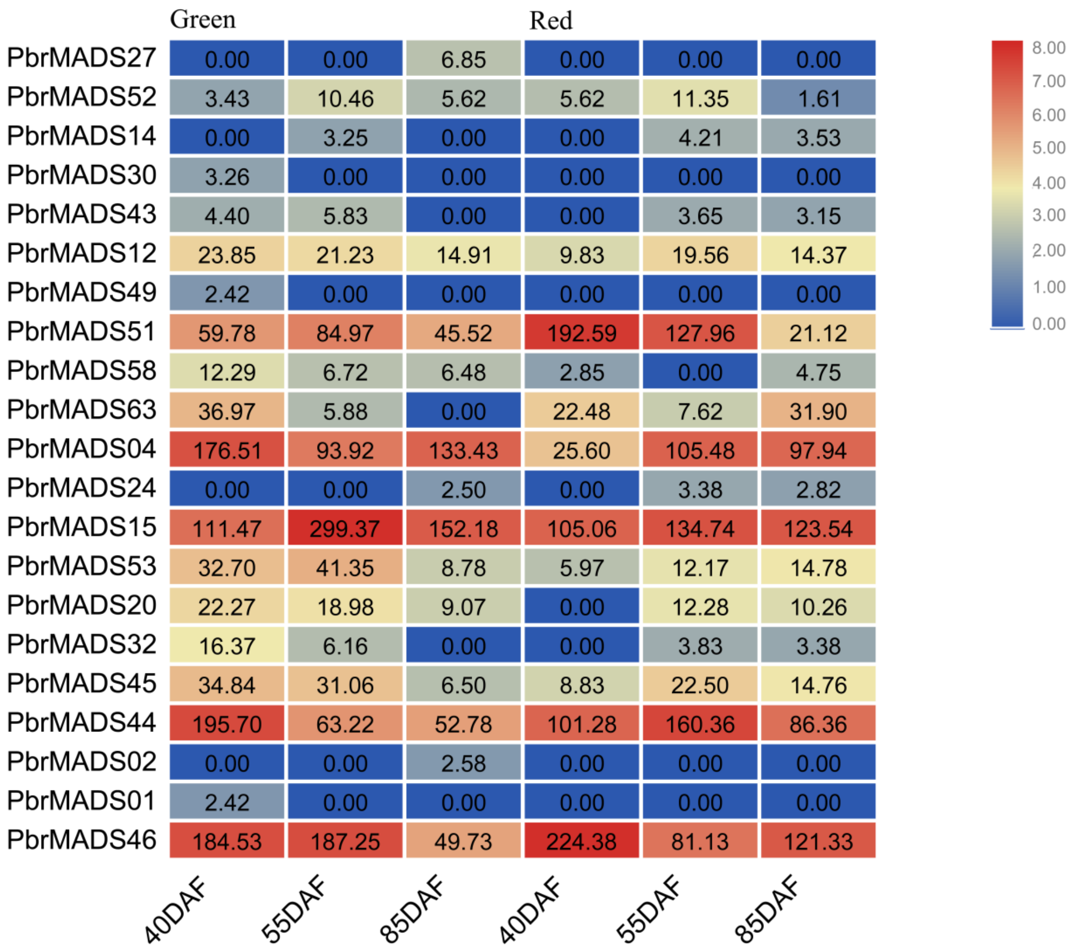

Fig.S13. Expression profiling of MADS-box divergent genes during three developmental stages in red/green skin color mutant of pear fruit. 40 DAF to 85 DAF indicated 40, 55 and 85 days after full blooming, respectively. These expression profile data were obtained using RNA-Seq data.

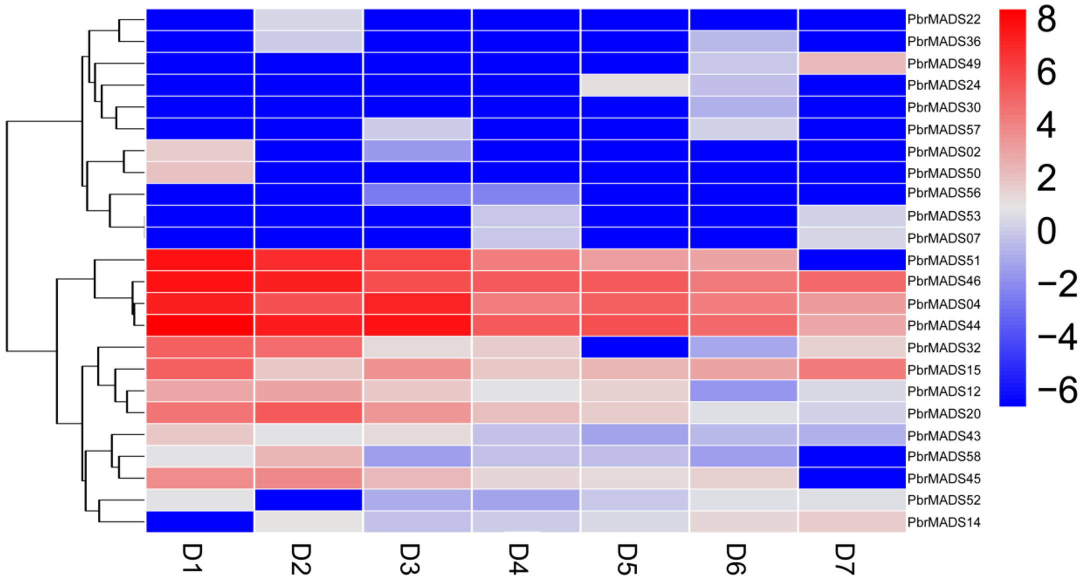

Fig.S14. Expression profiling of Chinese pear MADS-box genes during seven developmental stages of pear fruit. D 1 to D 5 indicated 15, 30, 55, 85 and 115 days after full blooming, respectively. D 6 indicated mature stage. D 7 indicated fruit senescence stage. These expression profile data were obtained using RNA-Seq data.

Table-S1: List of MADS-box genes identified in European pear and Chinese pear.

Name	Gene Identifier	Type	chr	Location COORDINATES (5′- 3′)	Introns	K domain (Y/N)	ORF length (bp)	Protein Length (a.a.)	Mol.Wt (Da)	PI
PcpMADS01	PCP032215.1	AGL13	1	9485328-9491628	6	Y	576	192	22508.89	9.28
PcpMADS02	PCP002223.1	SOC1	1	9542989-9547609	6	Y	756	252	28852.02	7.61
PcpMADS03	PCP005676.1	Mβ	2	14458837-14470801	10	N	3243	1081	122058.49	8.42
PcpMADS04	PCP010977.1	Mα	3	16253424-16254855	0	N	1044	348	39413.98	6.12
PcpMADS05	PCP022822.1	Mγ	4	2970427-2971107	0	N	501	167	19686.11	9.53
PcpMADS06	PCP002673.1	B	4	11630746-11633260	6	Y	702	234	27191.58	8.78
PcpMADS07	PCP006918.1	Bs	5	5528164-5536648	9	Y	2334	778	88756.81	6.98
PcpMADS08	PCP011955.1	Mα	5	6390843-6391970	0	N	720	240	27000.69	6.13
PcpMADS09	PCP018352.1	Mα	5	10847700-10849323	1	N	828	276	31389.54	6.98
PcpMADS10	PCP018351.1	Mα	5	10845728-10846433	0	N	504	168	18793.59	9.62
PcpMADS11	PCP010536.1	Mα	5	12274281-12294461	14	N	2793	931	104970.76	7.88
PcpMADS12	PCP039918.1	Mα	5	12278363-12279010	0	N	645	215	24435.99	9.43
PcpMADS13	PCP036865.1	Mβ	6	2452578-2454113	1	N	1080	360	40899.02	6.61
PcpMADS14	PCP021413.1	C/D	6	10690312-10692652	6	Y	549	183	21173.35	10.12
PcpMADS15	PCP032175.1	A	6	14320245-14324651	7	Y	765	255	29413.34	9.24
PcpMADS16	PCP007521.1	AGL15/18	7	15236398-15240628	6	Y	729	243	27402.01	5.51
PcpMADS17	PCP035343.1	B	8	672748-681675	11	Y	2982	994	111731.8	5.13
PcpMADS18	PCP007024.1	MIKC*	8	7662020-7665425	8	N	888	296	33363.42	5.39
PcpMADS19	PCP018702.1	B	8	9476213-9479280	5	Y	651	217	25270.57	8.89
PcpMADS20	PCP018235.1	Mβ	8	10897497-10898747	0	N	939	313	35095.45	6.34
PcpMADS21	PCP022935.1	SVP	8	13900910-13910407	5	Y	651	217	24533.08	6.37
PcpMADS22	PCP022936.1	SVP	8	13933270-13946341	6	Y	696	232	25990.86	6.86
PcpMADS23	PCP005825.1	SVP	8	14045976-14054581	6	Y	693	231	25787.79	8.57
PcpMADS24	PCP004033.1	Mγ	8	14177968-14178982	0	N	765	255	29140.4	9.45
PcpMADS25	PCP002331.1	AGL12	8	14357677-14379497	25	Y	2670	890	100262.03	8.57
PcpMADS26	PCP032236.1	C/D	8	14414002-14427043	16	Y	1980	660	74042.26	9.89
PcpMADS27	PCP002392.1	Mα	9	5277571-5296403	18	N	3207	1069	118885.59	7.24
PcpMADS28	PCP038375.1	Mα	9	5295600-5296301	0	N	699	233	26325.61	9.22
PcpMADS29	PCP029223.1	Mα	9	5840599-5844991	1	N	570	190	20822.98	9.96
PcpMADS30	PCP023681.1	C/D	9	6661243-6667959	6	Y	684	228	26254.57	9.63
PcpMADS31	PCP012204.1	E	9	7369246-7374848	7	Y	738	246	28071.88	8.16
PcpMADS32	PCP030812.1	Bs	9	11268597-11280017	7	N	717	239	27077.58	8.97

PcpMADS33	PCP006149.1	Bs	10	3512587-3522057	10	Y	1224	408	46406.43	9.25
PcpMADS34	PCP028978.1	Mα	10	6183301-6186885	0	N	681	227	25701.35	9.45
PcpMADS35	PCP028979.1	Mα	10	6189621-6190604	0	N	711	237	26329.25	9.2
PcpMADS36	PCP004454.1	Bs	10	11864290-11870132	6	Y	801	267	30936.03	6.79
PcpMADS37	PCP024342.1	Mα	10	16320432-16321112	0	N	525	175	19603.7	9.92
PcpMADS38	PCP024343.1	Mα	10	16322950-16323992	0	N	633	211	24198.19	7.12
PcpMADS39	PCP031198.1	C/D	10	16691041-16697853	5	Y	546	182	20727.68	9.66
PcpMADS40	PCP017259.1	Mα	11	18237744-18238638	0	N	555	185	20661.24	5.29
PcpMADS41	PCP021152.1	Mγ	12	693090-693924	0	N	639	213	24600.48	9.06
PcpMADS42	PCP023358.1	Mα	12	9274398-9275180	0	N	600	200	22754.2	6.97
PcpMADS43	PCP001807.1	A	13	2756142-2760364	6	Y	717	239	27984.79	7.66
PcpMADS44	PCP001808.1	E	13	2764229-2770504	7	Y	705	235	26537.18	8.36
PcpMADS45	PCP003345.1	MIKC*	13	3919125-3930551	10	N	1053	351	39569.91	7.11
PcpMADS46	PCP032202.1	Mγ	13	9244185-9244655	0	N	468	156	18149.12	9.52
PcpMADS47	PCP020055.1	E	14	1631267-1637665	7	Y	747	249	28519.18	6.67
PcpMADS48	PCP006689.1	MIKC*	14	4012409-4022156	14	N	2619	873	98484.93	5.67
PcpMADS49	PCP024843.1	AGL15/18	14	8472798-8478232	6	Y	756	252	28286.99	9.13
PcpMADS50	PCP014552.1	B	15	21553873-21556197	6	Y	711	237	27792.4	9.24
PcpMADS51	PCP023022.1	AGL12	15	24886878-24887927	2	N	432	144	16204.05	9.11
PcpMADS52	PCP016766.1	Mβ	15	26416374-26436140	9	N	4128	1376	153753.9	5.14
PcpMADS53	PCP034714.1	A	15	26712437-26714188	3	N	381	127	14143.52	9.76
PcpMADS54	PCP013106.1	E	16	3260037-3265900	7	Y	711	237	27094.94	9.4
PcpMADS55	PCP013107.1	A	16	3268107-3271546	6	Y	717	239	28046.99	8.72
PcpMADS56	PCP010918.1	Mα	17	9926429-9927634	0	N	702	234	26433.82	9.36
PcpMADS57	PCP029668.1	Mβ	17	13921608-13922952	0	N	1086	362	41692.53	8.87
PcpMADS58	PCP038145.1	C/D	scaffold	38515-42213	7	Y	672	224	25389.25	10.33
PcpMADS59	PCP007626.1	SOC1	scaffold	77986-84813	4	N	495	165	18981.71	9.3
PcpMADS60	PCP014073.1	SVP	scaffold	10572-15757	7	Y	681	227	25767.11	5.63
PcpMADS61	PCP026843.1	Mα	scaffold	33116-34297	0	N	702	234	25844.89	8.52
PcpMADS62	PCP013543.1	MIKC*	scaffold	34841-40767	8	N	918	306	34573.55	5.28
PcpMADS63	PCP036689.1	Mα	scaffold	14009-14527	0	N	516	172	20167.73	5.32
PcpMADS64	PCP027135.1	Mγ	scaffold	1246-2214	0	N	762	254	29065.37	9.35
PcpMADS65	PCP033235.1	Mγ	scaffold	36232-36651	0	N	417	139	16077.9	9.75
PcpMADS66	PCP018825.1	Mγ	scaffold	496-1654	0	N	627	209	23893.3	9.24
PcpMADS67	PCP042962.1	Mβ	scaffold	11385-11385	6	N	579	193	21750.03	7.71
PcpMADS68	PCP044935.1	C/D	NA	NA	6	Y	735	245	27884.41	9.46
PcpMADS69	PCP044772.1	A	NA	NA	3	Y	639	213	24887.79	9.94
PcpMADS70	PCP044137.1	MIKC*	NA	NA	13	N	2496	832	94391.01	6.41
PcpMADS71	PCP044475.1	Mβ	NA	NA	2	N	1035	345	39408.5	5.86
PcpMADS72	PCP045086.1	Mγ	NA	NA	0	N	720	240	27389.41	9.42

PcpMADS73	PCP032233.1	AGL12	scaffold	97864-104017	6	Y	651	217	24898.02	8.66
PbrMADS01	Pbr032788.1	SOC1	1	8132734-8141421	6	Y	705	235	27075.83	9.05
PbrMADS02	Pbr032787.2	SOC1	1	8152105-8168611	6	Y	759	253	28991.31	8.69
PbrMADS03	Pbr018829.1	Mα	2	237735-238322	0	N	585	195	22136.29	8.41
PbrMADS04	Pbr018801.2	AGL13	2	603223-614105	16	Y	2001	667	73113.69	7.56
PbrMADS05	Pbr022939.1	Mβ	2	6904265-6904744	0	N	477	159	18469.46	9.87
PbrMADS06	Pbr022918.2	AGL15/18	2	7101509-7106026	7	Y	771	257	29230.06	6.53
PbrMADS07	Pbr040541.1	B	2	15725115-15727317	6	Y	702	234	27169.52	8.47
PbrMADS08	Pbr025860.1	AGL15/18	3	2235923-2242424	9	Y	966	322	36782.58	9.01
PbrMADS09	Pbr031473.1	Mα	3	9785284-9786327	0	N	1041	347	39127.52	6.12
PbrMADS10	Pbr006693.1	Mγ	4	2423936-2424439	0	N	501	167	19683.02	9.36
PbrMADS11	Pbr034610.1	Mα	5	7051644-7052366	0	N	720	240	26902.6	6.2
PbrMADS12	Pbr000556.1	C/D	5	24523577-24531982	7	Y	735	245	27948.6	9.48
PbrMADS13	Pbr017715.1	C/D	6	437472-439619	6	Y	597	199	22504.78	10
PbrMADS14	Pbr011423.3	MIKC*	6	1824609-1827345	8	N	1122	374	41995.3	6.32
PbrMADS15	Pbr020185.1	E	6	4424778-4429823	7	Y	744	248	28212.74	7.75
PbrMADS16	Pbr001551.1	SOC1	6	14880923-14887496	2	N	411	137	15526.73	9.59
PbrMADS17	Pbr006798.1	Mγ	6	18614500-18615171	0	N	669	223	25424.42	9.72
PbrMADS18	Pbr006795.1	Mγ	6	18621477-18621950	0	N	471	157	18386.39	9.78
PbrMADS19	Pbr006794.1	Mγ	6	18630739-18631368	0	N	627	209	23911.27	9.24
PbrMADS20	Pbr023545.1	E	6	21412278-21417880	7	Y	717	239	27447.08	8.89
PbrMADS21	Pbr009670.1	AGL15/18	7	1511095-1515332	7	Y	771	257	29102.88	5.59
PbrMADS22	Pbr013902.1	SOC1	7	12939336-12970807	7	Y	714	238	27165.8	8.2
PbrMADS23	Pbr019318.1	Mγ	8	641097-641097	0	N	765	255	29184.45	9.45
PbrMADS24	Pbr019340.1	SVP	8	967796-978558	7	Y	699	233	26342.37	8.3
PbrMADS25	Pbr026551.1	Mβ	8	4050787-4051728	0	N	939	313	35068.34	6.2
PbrMADS26	Pbr004234.1	AGL12	8	5488881-5495600	6	Y	606	202	23166.98	8.43
PbrMADS27	Pbr004263.1	Mγ	8	5777631-5778242	0	N	609	203	23363.64	8.32
PbrMADS28	Pbr032195.1	Mβ	8	6410894-6411958	0	N	1062	354	40724.42	8.75
PbrMADS29	Pbr035294.1	B	8	7359228-7362459	6	Y	645	215	24929.28	8.91
PbrMADS30	Pbr022012.1	MIKC*	8	12737277-12740508	8	N	906	302	34167.36	5.67
PbrMADS31	Pbr027548.1	Mα	9	10296082-10296783	0	N	699	233	26301.59	9.33
PbrMADS32	Pbr029686.2	C/D	9	13855887-13862738	7	Y	726	242	28085.73	9.53
PbrMADS33	Pbr021448.1	Bs	10	2035089-2037780	6	Y	777	259	29882.82	6.41
PbrMADS34	Pbr039562.1	Mα	10	6671771-6672298	0	N	525	175	19534.59	9.84
PbrMADS35	Pbr039561.1	Mα	10	6674815-6675441	0	N	624	208	23813.8	7.08
PbrMADS36	Pbr039503.1	C/D	10	7101939-7110441	7	Y	729	243	27847.55	9.43
PbrMADS37	Pbr030435.1	Mγ	10	15484934-15485575	0	N	639	213	24794.5	8.82
PbrMADS38	Pbr025657.1	Mα	10	16496114-16496827	0	N	711	237	26295.23	9.2
PbrMADS39	Pbr025656.1	Mα	10	16499696-16500379	0	N	681	227	25668.27	9.32

PbrMADS40	Pbr026073.1	Mγ	12	3868978-3869538	0	N	558	186	21882.5	9.32
PbrMADS41	Pbr026074.1	Mγ	12	3872966-3873643	0	N	675	225	26130.45	9.1
PbrMADS42	Pbr026075.1	Mγ	12	3878308-3878949	0	N	639	213	24580.42	9.12
PbrMADS43	Pbr039074.1	MIKC*	13	2703781-2707742	11	N	1311	437	49445.3	8.68
PbrMADS44	Pbr029990.1	A	13	4377373-4381393	6	Y	717	239	28027.9	7.66
PbrMADS45	Pbr029989.1	E	13	4385598-4392040	7	Y	918	306	34142.78	8.82
PbrMADS46	Pbr035643.1	E	13	5802860-5807937	7	Y	717	239	27328.09	8.53
PbrMADS47	Pbr003650.1	SVP	13	10532036-10535566	6	Y	672	224	25510.86	5.63
PbrMADS48	Pbr010321.1	Mγ	14	1878629-1879402	0	N	771	257	29192.2	8.27
PbrMADS49	Pbr002033.1	AGL15/18	14	7643633-7648945	7	Y	798	266	29999.92	8.98
PbrMADS50	Pbr036879.1	C/D	14	14005515-14009216	6	Y	669	223	25327.33	10.69
PbrMADS51	Pbr007180.1	A	14	15049496-15055088	7	Y	765	255	29453.35	9.31
PbrMADS52	Pbr007292.1	MIKC*	14	15828084-15836608	16	N	2424	808	91387.26	6.49
PbrMADS53	Pbr022146.1	B	15	19423131-19425153	6	Y	720	240	28182.79	9.13
PbrMADS54	Pbr039693.1	SVP	15	26891365-26894987	6	Y	672	224	25305.69	6.48
PbrMADS55	Pbr042160.2	MIKC*	15	33038479-33044560	11	N	1041	347	39242.83	5.29
PbrMADS56	Pbr000828.1	C/D	15	40714954-40721950	7	Y	672	224	25827.56	9.45
PbrMADS57	Pbr000804.1	AGL12	15	40944071-40950751	5	Y	564	188	21510.99	7.7
PbrMADS58	Pbr015153.1	E	16	5932222-5938012	7	Y	750	250	28457.08	7.65
PbrMADS59	Pbr005989.1	Mγ	16	10115643-10116284	0	N	639	213	24580.42	9.12
PbrMADS60	Pbr005990.1	Mγ	16	10121071-10121748	0	N	675	225	26088.37	9.1
PbrMADS61	Pbr005991.1	Mγ	16	10125235-10125795	0	N	558	186	21882.54	9.4
PbrMADS62	Pbr037101.1	Mβ	17	4143639-4144730	0	N	1089	363	41803.72	8.96
PbrMADS63	Pbr016599.2	A	17	13722980-13723684	4	Y	663	221	25806.76	9.7
PbrMADS64	Pbr033418.1	Mα	17	13872712-13873416	0	N	702	234	26314.66	9.28
PbrMADS65	Pbr033409.1	Mα	17	17667140-17673579	0	N	702	234	26314.66	9.28
PbrMADS66	Pbr003216.1	Mβ	scaffold	13074-14075	0	N	999	333	37171.97	6.14
PbrMADS67	Pbr025970.1	Mα	scaffold	29167-29871	0	N	702	234	25806.89	8.51
PbrMADS68	Pbr036986.1	Mγ	scaffold	65837-66163	0	N	324	108	12710.85	9.82
PbrMADS69	Pbr001457.1	SOC1	scaffold	93002-97487	5	Y	714	238	27117.08	8.19
PbrMADS70	Pbr001458.1	SOC1	scaffold	108284-109564	2	N	348	116	13371.33	8.93
PbrMADS71	Pbr001460.1	SOC1	scaffold	121179-122459	2	N	348	116	13370.39	9.33
PbrMADS72	Pbr009640.1	Mγ	scaffold	126297-127616	0	N	1317	439	48527.26	5.28
PbrMADS73	Pbr036992.1	Mγ	scaffold	145135-145461	0	N	324	108	12710.85	9.82
PbrMADS74	Pbr025981.1	Mα	scaffold	377815-378519	0	N	702	234	25806.89	8.51

Table S2: All MEME motif sequences in European pear and Chinese pear MADS-box proteins.

Motif	Best possible match	Best possible match
1	41	MGRGKIEIKRIENKTNRQVTFSKRRNGLFKKAYELSVLCDA
2	21	EVAVIVFSPTGKLFEFCHPSV
3	33	MLGEDLDCLDMDELQQLEHQLETGLKRIRSRKT
4	73	ACAIIYSPYDTQPEVWPCPDGVQRIIAKFKTMPEMEQSKKMV NQESFLRQRIEKAQEQLKKQKKENREKEMTI
5	101	GTMQLIEAHRNATVHELNTELTQVMNQLEAEKKRGDQLNQ MTRVSQAQCWWEAPVDEMQMPQLDQLKSSLVDLKMNVTK QADRVLIQNTLNPNSQFFVGSS
6	200	PAELLEIDLVLRDQIPVIRRFTGGGTVTVDQNTLFVTFICNKD GVPGLQPYPRPIMSWSSLVYSKVFEGLADFQLRENDYVFGN RKFGGNAQSISKNRWIHHTSFLWDYDVRNMAYLKHPKRVP EYRLARDHLEFICRMKDYIPRSIFLEKTVEALGTQFSVRPERS DAIEATSNTKFVPSTRLLTRQELEEAAAFDSQA
7	171	QEAQHITWLFNGDNQHMIFPNEPNYLPHRGVECSTNATVPH YTNYFGTGTSTSPSTGKQSEAGDPVQLDTMGQISNMEGGGG FNEFDINACLSTEPGEHYAYPTYCSSYVPDDQKVKHEMEMNI PANPVDYQLSSNFELPRSLYENDHHAWLSSPGPSGIAVYKEN TYQPV
8	21	WYQEYAKLKAKVEVLQRTQRH
9	24	QIWHEQIEELQNKEWMLQEANNYL
10	40	CSYGWWERPIGMMTSLEELKEYKDALYKLKHNVEIRLDEM
11	57	TFLLPAGQSDNHLQNNNIHHSSNKKQIEDYVEASRRLKEAKV LKGGKKNNSNNNGGD
12	39	RKHICEEKYPTWDDRIDAFSEDQLIDLLAALEAKIQAGH
13	11	DAIIDRYQKHC
14	57	KQRMVMLSARGDIGPAAIMELENLNNVGEEGVTSESATNVT ICSSSALSLEDDCSDI
15	77	QAHHEAHEQVENLEMIPKSEEHRHVLDQFPFGGEEQPSSVLQ LATLQPQPQFNPYPHQPDHHHPGLLDFNLSGPSMY
16	29	PLHGLDMVDLNDLGWMIDHCVKEINNRMK
17	30	MDKAMNDGFGCSPVSLVALLLVLIILNWLIVLHFNSNLRYLF LCFRCDVMQIKPVCQRSCTDEKVCDHKPSEGVEINSIT
18	57	QPQVQNWEQQNHGLDLLPQPLPCLNNGGTQQDEFLQVRRN QLDLTLEPFYECHLGCF
19	57	DVCMIIYGPKQTGRSPELHTWPKNPEEVNRIINKYKASTMCK PAKKTFDLSDLLMDR
20	32	PQERAKRKLESLEVLKKTFKKLDHDVNVQDFM

Table-S3: Environmental selection pressure analysis of MADS-box genes in European pear and Chinese pear.

Paralogous Pairs		Ks	Ka	Ka/Ks
PcpMADS68	PcpMADS39	0.1189	0.0406	0.341463415
PcpMADS58	PcpMADS14	0.2504	0.1177	0.470047923
PcpMADS49	PcpMADS16	2.3565	0.3986	0.169149162
PcpMADS59	PcpMADS02	2.1844	0.4318	0.197674419
PcpMADS51	PcpMADS25	0.4558	0.163	0.357612988
PcpMADS07	PcpMADS36	0.184	0.0654	0.355434783
PcpMADS55	PcpMADS43	0.1255	0.0328	0.261354582
PcpMADS33	PcpMADS32	0.5694	0.3362	0.590446084
PcpMADS31	PcpMADS47	1.2324	0.2968	0.240830899
PcpMADS44	PcpMADS54	0.1121	0.0404	0.360392507
PcpMADS50	PcpMADS06	0.1619	0.0378	0.233477455
PcpMADS70	PcpMADS48	0.217	0.0751	0.346082949
PcpMADS62	PcpMADS18	0.80509	0.5563	0.690978648
PcpMADS03	PcpMADS57	0.8587	0.2031	0.236520321
PcpMADS20	PcpMADS52	0.4536	0.164	0.361552028
PcpMADS71	PcpMADS13	0.3733	0.1349	0.361371551
PcpMADS46	PcpMADS65	0.2494	0.0962	0.385725742
PcpMADS41	PcpMADS05	0.5709	0.2572	0.450516728
PcpMADS24	PcpMADS64	0.2386	0.053	0.222129086
PcpMADS66	PcpMADS72	0.9537	0.2336	0.244940757
PcpMADS28	PcpMADS27	0.188	0.0793	0.421808511
PcpMADS09	PcpMADS38	0.317	0.1222	0.385488959
PcpMADS10	PcpMADS37	0.0871	0.0383	0.439724455
PcpMADS12	PcpMADS11	0.0893	0.0668	0.748040314
PcpMADS63	PcpMADS40	0.8284	0.3262	0.393771125
PbrMADS40	PbrMADS59	0.4574	0.1534	0.335373852
PbrMADS52	PbrMADS14	0.2327	0.0899	0.386334336
PbrMADS14	PbrMADS43	1.6102	0.4198	0.260712955
PbrMADS31	PbrMADS65	0.1665	0.0611	0.366966967
PbrMADS28	PbrMADS62	0	0.0012	NA
PbrMADS65	PbrMADS64	0	0	NA
PbrMADS12	PbrMADS36	0.0924	0.0342	0.37012987
PbrMADS57	PbrMADS26	0.2259	0.0532	0.235502435
PbrMADS51	PbrMADS44	1.7747	0.241	0.1357976
PbrMADS21	PbrMADS06	0.1546	0.0341	0.220569211
PbrMADS22	PbrMADS02	0.2557	0.1737	0.679311693
PbrMADS58	PbrMADS46	0.1284	0.0219	0.170560748

PbrMADS63	PbrMADS51	1.7706	0.4394	0.248164464
PbrMADS13	PbrMADS50	0.2211	0.142	0.642243329
PbrMADS15	PbrMADS51	2.8424	0.5709	0.200851393
PbrMADS53	PbrMADS07	0.1446	0.0373	0.257952974
PbrMADS32	PbrMADS36	1.6039	0.2259	0.140844192
PbrMADS45	PbrMADS15	1.9994	0.3141	0.157097129
PbrMADS44	PbrMADS63	3.1555	0.4311	0.136618602

Table-S4: Orthologous analyses of MADS-box genes between European pear and Chinese pear.

Numble	orthologous genes		Ks	Ka	Ka/Ks	r
segdup00001	PcpMADS02	PbrMADS69	0.0132	0.0126	0.954545455	NA
segdup00002	PcpMADS36	PbrMADS33	0.0262	0.0058	0.221374046	NA
segdup00003	PcpMADS73	PbrMADS26	0.0236	0.0022	0.093220339	NA
segdup00004	PcpMADS14	PbrMADS13	0.1025	0.0768	0.749268293	NA
segdup00005	PcpMADS58	PbrMADS50	0.0274	0.0084	0.306569343	0.870488042
segdup00006	PcpMADS30	PbrMADS32	0.0262	0.0057	0.217557252	0.546106133
segdup00007	PcpMADS26	PbrMADS56	0.2181	0.1471	0.674461256	-0.4805133
segdup00008	PcpMADS68	PbrMADS12	0.0293	0.013	0.443686007	0.007729643
segdup00009	PcpMADS59	PbrMADS16	0.011	0	0	NA
segdup00010	PcpMADS43	PbrMADS44	0.033	0.0054	0.163636364	0.710371262
segdup00011	PcpMADS15	PbrMADS51	0.1509	0.0649	0.43008615	0.592274491
segdup00012	PcpMADS69	PbrMADS63	0.0346	0	0	NA
segdup00013	PcpMADS01	PbrMADS04	1.732	0.3496	0.201847575	NA
segdup00014	PcpMADS44	PbrMADS45	0.0339	0.0079	0.233038348	0.643724157
segdup00015	PcpMADS47	PbrMADS15	0.0949	0.0885	0.93256059	0.048385689
segdup00016	PcpMADS31	PbrMADS20	0.1161	0.0273	0.235142119	0.346439237
segdup00017	PcpMADS50	PbrMADS53	0.0065	0	0	-0.356016289
segdup00018	PcpMADS06	PbrMADS07	0.02	0.0018	0.09	0.351224197
segdup00019	PcpMADS19	PbrMADS29	0.0289	0.0344	1.190311419	NA
segdup00020	PcpMADS60	PbrMADS47	0.0276	0.0136	0.492753623	NA
segdup00021	PcpMADS16	PbrMADS21	0.0581	0.0089	0.153184165	NA
segdup00022	PcpMADS18	PbrMADS30	0.0097	0.0029	0.298969072	0.702609363
segdup00023	PcpMADS62	PbrMADS55	0	0.0014	NA	NA
segdup00024	PcpMADS45	PbrMADS43	0.0166	0.0089	0.536144578	-0.476315247
segdup00025	PcpMADS48	PbrMADS52	0.0473	0.025	0.528541226	0.504859396
segdup00026	PcpMADS70	PbrMADS14	0.0677	0.0501	0.740029542	0.852425165
segdup00027	PcpMADS34	PbrMADS39	0	0.0057	NA	NA
segdup00028	PcpMADS35	PbrMADS38	0.0131	0.0018	0.13740458	NA
segdup00029	PcpMADS08	PbrMADS11	0.0689	0.0106	0.153846154	NA
segdup00030	PcpMADS38	PbrMADS35	0.0222	0.0021	0.094594595	NA

segdup00031	PcpMADS04	PbrMADS09	0.022	0.0099	0.45	NA
segdup00032	PcpMADS03	PbrMADS05	0.0185	0.0055	0.297297297	NA
segdup00033	PcpMADS20	PbrMADS25	0.0195	0.0041	0.21025641	NA
segdup00034	PcpMADS72	PbrMADS17	0.3089	0.0897	0.290385238	NA
segdup00035	PcpMADS66	PbrMADS19	0	0.004	NA	NA
segdup00036	PcpMADS24	PbrMADS23	0.0456	0.0033	0.072368421	NA
segdup00037	PcpMADS21	PbrMADS24	0.0141	0.002	0.141843972	0.56840007

Table-S5: Estimates of the Ks for the WGD events in European pear.

Paralogous Pairs		Number of conserved flanking protein-coding genes	Ka/Ks (mean ± s.d.)	Ks (mean ± s.d.)
PcpMADS68	PcpMADS39	10	0.3658 ± 0.2131	0.1788 ± 0.0758
PcpMADS58	PcpMADS14	7	0.1499 ± 0.0861	0.1872 ± 0.0146
PcpMADS49	PcpMADS16	5	0.4652 ± 0.1402	1.4838 ± 0.1802
PcpMADS59	PcpMADS02	4	0.8878 ± 0.2855	1.4937 ± 0.1901
PcpMADS51	PcpMADS25	8	0.3904 ± 0.2045	0.1316 ± 0.0356
PcpMADS07	PcpMADS36	5	1.2165 ± 0.3477	0.1909 ± 0.0789
PcpMADS55	PcpMADS43	7	0.2676 ± 0.1094	0.1763 ± 0.0675
PcpMADS33	PcpMADS32	3	1.2574 ± 0.1561	0.6305 ± 0.2107
PcpMADS31	PcpMADS47	3	0.6209 ± 0.1994	1.4522 ± 0.0561
PcpMADS44	PcpMADS54	3	0.2521 ± 0.0182	0.1869 ± 0.0443
PcpMADS50	PcpMADS06	4	0.2949 ± 0.1317	0.1654 ± 0.0108
PcpMADS70	PcpMADS48	4	0.2929 ± 0.1017	0.1651 ± 0.0069
PcpMADS62	PcpMADS18	4	1.5088 ± 0.4554	1.0729 ± 0.0348
PcpMADS03	PcpMADS57	6	0.3271 ± 0.1232	0.8502 ± 0.0483
PcpMADS20	PcpMADS52	3	0.2215 ± 0.0494	0.1642 ± 0.0386
PcpMADS71	PcpMADS13	5	0.4314 ± 0.0965	0.3391 ± 0.0299
PcpMADS46	PcpMADS65	3	0.4877 ± 0.0308	0.1854 ± 0.0011
PcpMADS41	PcpMADS05	6	0.5738 ± 0.2138	0.2349 ± 0.1009
PcpMADS24	PcpMADS64	5	0.3312 ± 0.1681	0.1606 ± 0.0058
PcpMADS66	PcpMADS72	3	0.3202 ± 0.0494	0.9099 ± 0.2127
PcpMADS09	PcpMADS38	7	0.3429 ± 0.1573	0.1742 ± 0.0341
PcpMADS10	PcpMADS37	4	0.7081 ± 0.2121	0.1367 ± 0.0324
PcpMADS63	PcpMADS40	5	0.4337 ± 0.1035	0.6365 ± 0.0339

We discarded any Ks values >2.0 because of the risk of saturation.

Table-S6: Estimates of the Ks for the WGD events in Chinese pear.

Paralogous Pairs		Number of conserved flanking protein-coding genes	Ka/Ks (mean ±s.d.)	Ks (mean ± s.d.)
PbrMADS40	PbrMADS59	6	0.3242 ±0.0933	0.3009 ±0.3628
PbrMADS52	PbrMADS14	13	0.2458 ±0.1161	0.2171 ±0.0894
PbrMADS14	PbrMADS43	3	0.3911 ±0.1237	1.1279 ±0.1145
PbrMADS31	PbrMADS65	4	0.3173 ±0.0645	0.1662 ±0.0259
PbrMADS28	PbrMADS62	2	0.8222 ±0.2383	0.0192 ±0.0094
PbrMADS65	PbrMADS64	4	0.1523 ±0.1016	0.0544 ±0.0778
PbrMADS12	PbrMADS36	4	0.3817 ±0.0221	0.0889 ±0.0052
PbrMADS57	PbrMADS26	5	0.2704 ±0.0573	0.1781 ±0.0352
PbrMADS51	PbrMADS44	2	0.5373 ±0.1745	1.5798 ±0.2618
PbrMADS21	PbrMADS06	7	0.2796 ±0.0988	0.1892 ±0.0261
PbrMADS22	PbrMADS02	8	0.3163 ±0.1831	0.2013 ±0.0555
PbrMADS58	PbrMADS46	12	0.3471 ±0.1686	0.2139 ±0.1077
PbrMADS63	PbrMADS51	4	0.5642 ±0.3842	1.7663 ±0.1111
PbrMADS13	PbrMADS50	13	0.3787 ±0.1897	0.1872 ±0.0455
PbrMADS15	PbrMADS51	5	0.4046 ±0.2309	1.8231 ±0.1411
PbrMADS53	PbrMADS07	5	0.3198 ±0.1904	0.1997 ±0.0627
PbrMADS32	PbrMADS36	4	0.2691 ±0.2187	1.2982 ±0.1595
PbrMADS45	PbrMADS15	4	0.8045 ±0.2112	1.3131 ±0.1365
PbrMADS44	PbrMADS63	5	0.4269 ±0.2414	1.7435 ±0.0352

We discarded any Ks values >2.0 because of the risk of saturation.

Table-S7: Estimates of the Ks for the WGD events between Chinese pear and European pear.

Orthologous Pairs		Number of conserved flanking protein-coding genes	Ka/Ks (mean ±s.d.)	Ks (mean ± s.d.)
PcpMADS02	PbrMADS69	4	0.9077 ±0.1441	0.0185 ±0.0099
PcpMADS36	PbrMADS33	3	0.2281 ±0.1215	0.0218 ±0.0038
PcpMADS14	PbrMADS13	7	0.7636 ±0.3288	0.0432 ±0.0291
PcpMADS58	PbrMADS50	7	0.3791 ±0.1813	0.0195 ±0.0064
PcpMADS30	PbrMADS32	5	0.3974 ±0.1634	0.0353 ±0.0189
PcpMADS26	PbrMADS56	10	0.3517 ±0.1969	0.1446 ±0.0395
PcpMADS68	PbrMADS12	3	0.2313 ±0.1237	0.0419 ±0.0203
PcpMADS59	PbrMADS16	15	0.3734 ±0.2941	0.1945 ±0.0589
PcpMADS43	PbrMADS44	5	0.2234 ±0.0678	0.0248 ±0.0059
PcpMADS15	PbrMADS51	6	0.3981 ±0.0961	0.1437 ±0.0117
PcpMADS69	PbrMADS63	5	0.3688 ±0.2405	0.0296 ±0.0084

PcpMADS01	PbrMADS04	3	0.5009±0.0126	1.7559±0.0311
PcpMADS44	PbrMADS45	5	0.3968±0.1745	0.0312±0.0084
PcpMADS47	PbrMADS15	10	0.3218±0.1869	0.1413±0.0308
PcpMADS31	PbrMADS20	2	0.2284±0.0766	0.1299±0.0239
PcpMADS50	PbrMADS53	4	0.6246±0.2691	0.0319±0.0214
PcpMADS06	PbrMADS07	5	0.3324±0.0921	0.0292±0.0162
PcpMADS19	PbrMADS29	11	0.3356±0.1897	0.0357±0.0278
PcpMADS60	PbrMADS47	3	0.4809±0.1246	0.0256±0.0024
PcpMADS16	PbrMADS21	5	0.2817±0.1797	0.0593±0.0388
PcpMADS18	PbrMADS30	8	0.5869±0.2417	0.0934±0.0779
PcpMADS62	PbrMADS55	5	0.7049±0.1803	0.1584±0.1926
PcpMADS45	PbrMADS43	4	0.7297±0.0883	0.0149±0.0089
PcpMADS48	PbrMADS52	5	0.2598±0.1539	0.0319±0.0177
PcpMADS70	PbrMADS14	14	0.3681±0.1796	0.0226±0.0085
PcpMADS34	PbrMADS39	6	0.4888±0.4601	0.0331±0.0262
PcpMADS35	PbrMADS38	7	0.4888±0.4601	0.0331±0.0262
PcpMADS08	PbrMADS11	5	0.2397±0.0524	0.0354±0.0193
PcpMADS38	PbrMADS35	3	0.2147±0.0448	0.0236±0.0048
PcpMADS04	PbrMADS09	3	0.1887±0.0203	0.0663±0.0031
PcpMADS03	PbrMADS05	4	0.6494±0.0803	0.0121±0.0067
PcpMADS20	PbrMADS25	12	0.2803±0.1563	0.0294±0.0145
PcpMADS72	PbrMADS17	6	0.2271±0.0354	0.2767±0.0272
PcpMADS66	PbrMADS19	5	0.2954±0.1552	0.0227±0.0052
PcpMADS24	PbrMADS23	5	0.2289±0.1311	0.0248±0.0119
PcpMADS21	PbrMADS24	4	0.2269±0.0593	0.0399±0.0121

We discarded any Ks values >2.0 because of the risk of saturation.

Table S8: Blast2GO annotation details of MADS-box protein sequences of European pear and Chinese pear.

Gene name	GO
PbrMADS60	GO:0005634,GO:0006355,GO:0046983,GO:0003677,GO:0006351
PbrMADS62	GO:0005634,GO:0006355,GO:0046983,GO:0003677,GO:0006351
PbrMADS61	GO:0005634,GO:0006355,GO:0046983,GO:0003677,GO:0006351
PbrMADS57	GO:0005634,GO:0046983,GO:0040008,GO:0055114,GO:0016491,GO:0048364,GO:0006351,GO:0006355,GO:0003700,GO:0010228,GO:0009908,GO:0016020,GO:0016021,GO:0032440,GO:0003677
PbrMADS13	GO:0005634,GO:0006355,GO:0003700,GO:0016020,GO:0016021,GO:0046983,GO:0003677,GO:0006351
PbrMADS12	GO:0005634,GO:0006355,GO:0003700,GO:0016020,GO:0016021,GO:0046983,

	GO:0005515,GO:0003677,GO:0006351
PbrMADS56	GO:0048481,GO:0005634,GO:0046983,GO:0005515,GO:0006351,GO:0006355,GO:0003700,GO:0016020,GO:0016021,GO:0048440,GO:0080155,GO:0048283,GO:0048316,GO:0003677
PbrMADS59	GO:0005634,GO:0006355,GO:0046983,GO:0003677,GO:0006351
PbrMADS15	GO:0005634,GO:0006355,GO:0003700,GO:0046983,GO:0048440,GO:0048441,GO:0048442,GO:0048443,GO:0005515,GO:0010076,GO:0003677,GO:0006351
PbrMADS14	GO:0005634,GO:0006355,GO:0009555,GO:0016020,GO:0016021,GO:0046983,GO:0032440,GO:0055114,GO:0016491,GO:0003677,GO:0006351
PbrMADS58	GO:0048833,GO:0048481,GO:0005634,GO:0006355,GO:0003700,GO:0046983,GO:0005515,GO:0001708,GO:0003677,GO:0010093,GO:0006351
PbrMADS53	GO:0005634,GO:0006355,GO:0003700,GO:0046983,GO:0005515,GO:0003677,GO:0006351
PbrMADS52	GO:0006464,GO:0005634,GO:0006355,GO:0009555,GO:0016020,GO:0016021,GO:0046983,GO:0032440,GO:0055114,GO:0016491,GO:0003677,GO:0006351
PbrMADS11	GO:0005634,GO:0006355,GO:0003700,GO:0046983,GO:0009960,GO:0003677,GO:0006351
PbrMADS55	GO:0005634,GO:0046983,GO:0010152,GO:0055114,GO:0005515,GO:0016491,GO:0006351,GO:0006355,GO:0009555,GO:0003700,GO:0032440,GO:0003677,GO:0080092
PbrMADS10	GO:0005634,GO:0006355,GO:0046983,GO:0003677,GO:0006351
PbrMADS54	GO:0005634,GO:0000900,GO:0045892,GO:0046983,GO:0007275,GO:0006351,GO:0009266,GO:0006355,GO:0003700,GO:0016020,GO:0016021,GO:0009910,GO:0048438,GO:0017148,GO:0003677
PbrMADS17	GO:0005634,GO:0006355,GO:0046983,GO:0003677,GO:0006351
PbrMADS16	GO:0010048,GO:0005634,GO:0006355,GO:0003700,GO:0009908,GO:0046983,GO:0005515,GO:0007275,GO:0003677,GO:0048510,GO:0006351
PbrMADS19	GO:0005634,GO:0006355,GO:0046983,GO:0003677,GO:0006351
PbrMADS18	GO:0005634,GO:0006355,GO:0046983,GO:0003677,GO:0006351
PbrMADS51	GO:0005634,GO:0006355,GO:0003700,GO:0046983,GO:0032440,GO:0010154,GO:0055114,GO:0016491,GO:0003677,GO:0010077,GO:0006351
PbrMADS50	GO:0005634,GO:0006355,GO:0003700,GO:0016020,GO:0016021,GO:0046983,GO:0003677,GO:0006351
PbrMADS02	GO:0005634,GO:0010150,GO:0046983,GO:0030154,GO:0055114,GO:0007275,GO:0016491,GO:0009838,GO:0006351,GO:0006355,GO:0003700,GO:0009908,GO:0009909,GO:0032440,GO:0080187,GO:0003677
PbrMADS46	GO:0048833,GO:0048481,GO:0005634,GO:0006355,GO:0003700,GO:0046983,GO:0005515,GO:0001708,GO:0003677,GO:0010093,GO:0006351
PbrMADS45	GO:0005634,GO:0006355,GO:0003700,GO:0046983,GO:0048440,GO:0048441,GO:0048442,GO:0048443,GO:0010076,GO:0003677,GO:0006351
PbrMADS01	GO:0005634,GO:0010150,GO:0046983,GO:0055114,GO:0016491,GO:0009838,GO:0006351,GO:0006355,GO:0003700,GO:0009909,GO:0032440,GO:0080187,

	GO:0003677
PbrMADS48	GO:0005634,GO:0006355,GO:0046983,GO:0003677,GO:0006351
PbrMADS04	GO:0008865,GO:0016740,GO:0016773,GO:0005829,GO:0000166,GO:0004747,GO:0016301,GO:0005524,GO:0019252,GO:0016310,GO:0005975,GO:0006014,GO:0046686
PbrMADS03	GO:0005634,GO:0006355,GO:0003700,GO:0043078,GO:0046983,GO:0009559,GO:0005515,GO:0003677,GO:0006351
PbrMADS47	GO:0005634,GO:0000900,GO:0045892,GO:0046983,GO:0007275,GO:0006351,GO:0009266,GO:0006355,GO:0003700,GO:0009910,GO:0048438,GO:0017148,GO:0003677
PbrMADS42	GO:0005634,GO:0006355,GO:0046983,GO:0003677,GO:0006351
PbrMADS41	GO:0005634,GO:0006355,GO:0046983,GO:0003677,GO:0006351
PbrMADS44	GO:0005634,GO:0006355,GO:0003700,GO:0009908,GO:0046983,GO:0030154,GO:0007275,GO:0003677,GO:0006351
PbrMADS43	GO:0005634,GO:0006355,GO:0003700,GO:0046983,GO:0003677,GO:0006351
PbrMADS09	GO:0005634,GO:0006355,GO:0016020,GO:0016021,GO:0046983,GO:0003677,GO:0006351
PbrMADS06	GO:0009793,GO:0048577,GO:0005634,GO:0060867,GO:0045892,GO:0045893,GO:0046983,GO:0005737,GO:0006351,GO:0006355,GO:0042803,GO:0010227,GO:0003700,GO:0009910,GO:0010262,GO:0003677,GO:0060862,GO:0010047
PbrMADS05	GO:0005634,GO:0006355,GO:0046983,GO:0003677,GO:0006351
PbrMADS49	GO:0009793,GO:0005634,GO:0060867,GO:0045892,GO:0045893,GO:0046983,GO:0005737,GO:0006351,GO:0006355,GO:0042803,GO:0010227,GO:0003700,GO:0009910,GO:0010262,GO:0003677,GO:0060862,GO:0010047
PbrMADS08	GO:0009793,GO:0005634,GO:0060867,GO:0045892,GO:0045893,GO:0046983,GO:0005737,GO:0006351,GO:0006355,GO:0042803,GO:0010227,GO:0003700,GO:0009910,GO:0010262,GO:0003677,GO:0060862,GO:0010047
PbrMADS07	GO:0005634,GO:0006355,GO:0003700,GO:0046983,GO:0005515,GO:0003677,GO:0006351
PbrMADS40	GO:0005634,GO:0006355,GO:0046983,GO:0003677,GO:0006351
PbrMADS35	GO:0005634,GO:0006355,GO:0046983,GO:0003677,GO:0006351
PbrMADS34	GO:0005634,GO:0006355,GO:0003700,GO:0046983,GO:0003677,GO:0006351
PbrMADS37	GO:0005634,GO:0006355,GO:0046983,GO:0003677,GO:0006351
PbrMADS36	GO:0005634,GO:0006355,GO:0003700,GO:0016020,GO:0016021,GO:0046983,GO:0005515,GO:0003677,GO:0006351
PbrMADS31	GO:0005634,GO:0006355,GO:0046983,GO:0003677,GO:0006351
PbrMADS30	GO:0005634,GO:0006355,GO:0003700,GO:0016020,GO:0016021,GO:0046983,GO:0032440,GO:0055114,GO:0016491,GO:0003677,GO:0006351
PbrMADS74	GO:0005634,GO:0006355,GO:0046983,GO:0003677,GO:0006351
PbrMADS33	GO:0005634,GO:0006355,GO:0003700,GO:0046983,GO:0032440,GO:0055114,GO:0016491,GO:0003677,GO:0006351
PbrMADS32	GO:0005634,GO:0006355,GO:0003700,GO:0046983,GO:0003677,GO:0006351

PbrMADS39	GO:0005634,GO:0006355,GO:0003700,GO:0046983,GO:0009960,GO:0003677,GO:0006351
PbrMADS38	GO:0005634,GO:0006355,GO:0016020,GO:0016021,GO:0046983,GO:0003677,GO:0006351
PbrMADS71	GO:0005634,GO:0010150,GO:0046983,GO:0055114,GO:0016491,GO:0009838,GO:0006351,GO:0006355,GO:0003700,GO:0009909,GO:0032440,GO:0080187,GO:0003677
PbrMADS70	GO:0005634,GO:0010150,GO:0046983,GO:0055114,GO:0016491,GO:0009838,GO:0006351,GO:0006355,GO:0003700,GO:0009909,GO:0032440,GO:0080187,GO:0003677
PbrMADS73	GO:0005634,GO:0006355,GO:0003700,GO:0046983,GO:0003677,GO:0006351
PbrMADS72	GO:0005634,GO:0006355,GO:0046983,GO:0003677,GO:0006351
PbrMADS68	GO:0005634,GO:0006355,GO:0003700,GO:0046983,GO:0003677,GO:0006351
PbrMADS24	GO:0005634,GO:0006355,GO:0003700,GO:0046983,GO:0003677,GO:0006351
PbrMADS23	GO:0005634,GO:0006355,GO:0046983,GO:0003677,GO:0006351
PbrMADS67	GO:0005634,GO:0006355,GO:0046983,GO:0003677,GO:0006351
PbrMADS26	GO:0005634,GO:0046983,GO:0040008,GO:0055114,GO:0016491,GO:0048364,GO:0006351,GO:0006355,GO:0003700,GO:0010228,GO:0009908,GO:0016020,GO:0016021,GO:0032440,GO:0003677
PbrMADS25	GO:0005634,GO:0006355,GO:0046983,GO:0003677,GO:0006351
PbrMADS69	GO:0005634,GO:0010150,GO:0046983,GO:0030154,GO:0055114,GO:0007275,GO:0016491,GO:0009838,GO:0006351,GO:0006355,GO:0003700,GO:0009908,GO:0009909,GO:0032440,GO:0080187,GO:0003677
PbrMADS64	GO:0005634,GO:0006355,GO:0046983,GO:0003677,GO:0006351
PbrMADS20	GO:0005634,GO:0006355,GO:0003700,GO:0046983,GO:0003677,GO:0006351
PbrMADS63	GO:0005634,GO:0006355,GO:0003700,GO:0046983,GO:0003677,GO:0006351
PbrMADS66	GO:0005634,GO:0006355,GO:0046983,GO:0003677,GO:0006351
PbrMADS22	GO:0005634,GO:0008134,GO:0010150,GO:0046983,GO:0005737,GO:0030154,GO:0055114,GO:0007275,GO:0016491,GO:0009838,GO:0006351,GO:0006355,GO:0003700,GO:0016020,GO:0009908,GO:0016021,GO:0009909,GO:0009911,GO:0032440,GO:0080187,GO:0000060,GO:0003677,GO:0010077
PbrMADS65	GO:0005634,GO:0006355,GO:0046983,GO:0003677,GO:0006351
PbrMADS21	GO:0009793,GO:0048577,GO:0005634,GO:0060867,GO:0045892,GO:0045893,GO:0046983,GO:0005737,GO:0005515,GO:0006351,GO:0006355,GO:0042803,GO:0010227,GO:0003700,GO:0009908,GO:0009910,GO:0010262,GO:0003677,GO:0060862,GO:0010047
PbrMADS28	GO:0005634,GO:0006355,GO:0046983,GO:0003677,GO:0006351
PbrMADS27	GO:0005634,GO:0006355,GO:0046983,GO:0003677,GO:0006351
PbrMADS29	GO:0005634,GO:0006355,GO:0003700,GO:0046983,GO:0032440,GO:0005737,GO:0055114,GO:0007275,GO:0016491,GO:0003677,GO:0010093,GO:0006351
PcpMADS35	GO:0005634,GO:0006355,GO:0016020,GO:0016021,GO:0046983,GO:0003677,GO:0006351

PcpMADS36	GO:0005634,GO:0006355,GO:0003700,GO:0046983,GO:0032440,GO:0055114,GO:0016491,GO:0003677,GO:0006351
PcpMADS33	GO:0048481,GO:0048833,GO:0005634,GO:0046983,GO:0030154,GO:0007275,GO:0001708,GO:0010093,GO:0006351,GO:0006355,GO:0003700,GO:0009908,GO:0016020,GO:0016021,GO:0048440,GO:0048441,GO:0048442,GO:0048443,GO:0010076,GO:0003677
PcpMADS34	GO:0005634,GO:0006355,GO:0046983,GO:0003677,GO:0006351
PcpMADS39	GO:0005634,GO:0006355,GO:0003700,GO:0046983,GO:0005515,GO:0003677,GO:0006351
PcpMADS37	GO:0005634,GO:0006355,GO:0003700,GO:0046983,GO:0003677,GO:0006351
PcpMADS38	GO:0005634,GO:0006355,GO:0046983,GO:0003677,GO:0006351
PcpMADS42	GO:0005634,GO:0006355,GO:0046983,GO:0003677,GO:0006351
PcpMADS43	GO:0005634,GO:0006355,GO:0003700,GO:0046983,GO:0007275,GO:0003677,GO:0006351
PcpMADS40	GO:0005634,GO:0006355,GO:0016020,GO:0003700,GO:0016021,GO:0043078,GO:0046983,GO:0009559,GO:0005515,GO:0003677,GO:0006351
PcpMADS41	GO:0005634,GO:0006355,GO:0046983,GO:0003677,GO:0006351
PcpMADS08	GO:0005634,GO:0006355,GO:0003700,GO:0046983,GO:0009960,GO:0003677,GO:0006351
PcpMADS09	GO:0005634,GO:0006355,GO:0046983,GO:0003677,GO:0006351
PcpMADS02	GO:0005634,GO:0008134,GO:0010150,GO:0046983,GO:0005737,GO:0055114,GO:0016491,GO:0009838,GO:0006351,GO:0006355,GO:0003700,GO:0016020,GO:0016021,GO:0009909,GO:0009911,GO:0032440,GO:0080187,GO:0000060,GO:0003677,GO:0010077
PcpMADS46	GO:0005634,GO:0006355,GO:0046983,GO:0003677,GO:0006351
PcpMADS47	GO:0005634,GO:0006355,GO:0003700,GO:0046983,GO:0048440,GO:0048441,GO:0048442,GO:0048443,GO:0005515,GO:0010076,GO:0003677,GO:0006351
PcpMADS03	GO:0009331,GO:0005829,GO:0051287,GO:0005975,GO:0016616,GO:0006072,GO:0046168,GO:0055114,GO:0016491,GO:0004367
PcpMADS44	GO:0005634,GO:0006355,GO:0003700,GO:0046983,GO:0048440,GO:0048441,GO:0048442,GO:0048443,GO:0010076,GO:0003677,GO:0006351
PcpMADS01	GO:0005634,GO:0045893,GO:0010022,GO:0046983,GO:0030154,GO:0055114,GO:0005515,GO:0007275,GO:0016491,GO:0010093,GO:0006351,GO:0006355,GO:0003700,GO:0009908,GO:0032440,GO:0003677
PcpMADS45	GO:0005634,GO:0006355,GO:0003700,GO:0046983,GO:0003677,GO:0006351
PcpMADS06	GO:0005634,GO:0006355,GO:0003700,GO:0046983,GO:0005515,GO:0003677,GO:0006351
PcpMADS07	GO:0045489,GO:0071555,GO:0005794,GO:0016020,GO:0016740,GO:0016021,GO:0016757,GO:0005768,GO:0005802,GO:0005515,GO:0000139
PcpMADS48	GO:0006464,GO:0005634,GO:0006355,GO:0009555,GO:0046983,GO:0032440,GO:0016874,GO:0055114,GO:0016491,GO:0003677,GO:0006351
PcpMADS04	GO:0005634,GO:0006355,GO:0046983,GO:0003677,GO:0006351

PcpMADS49	GO:0048577,GO:0009793,GO:0005634,GO:0060867,GO:0045892,GO:0045893,GO:0046983,GO:0005737,GO:0006351,GO:0006355,GO:0009555,GO:0042803,GO:0010227,GO:0003700,GO:0009910,GO:0010262,GO:0003677,GO:0060862,GO:0010047
PcpMADS05	GO:0005634,GO:0006355,GO:0046983,GO:0003677,GO:0006351
PcpMADS50	GO:0005634,GO:0006355,GO:0003700,GO:0046983,GO:0005515,GO:0003677,GO:0006351
PcpMADS53	GO:0005634,GO:0006355,GO:0003700,GO:0046983,GO:0003677,GO:0006351
PcpMADS10	GO:0005634,GO:0006355,GO:0003700,GO:0046983,GO:0003677,GO:0006351
PcpMADS54	GO:0005634,GO:0006355,GO:0003700,GO:0046983,GO:0048440,GO:0048441,GO:0048442,GO:0048443,GO:0010076,GO:0003677,GO:0006351
PcpMADS51	GO:0005634,GO:0046983,GO:0055114,GO:0016491,GO:0048364,GO:0006351,GO:0006355,GO:0003700,GO:0010228,GO:0016020,GO:0016021,GO:0032440,GO:0003677
PcpMADS52	GO:0016020,GO:0016021,GO:0016310,GO:0032440,GO:0055114,GO:0016491,GO:0016301
PcpMADS19	GO:0005634,GO:0006355,GO:0003700,GO:0046983,GO:0032440,GO:0005737,GO:0055114,GO:0016491,GO:0003677,GO:0010093,GO:0006351
PcpMADS13	GO:0005634,GO:0006355,GO:0046983,GO:0003677,GO:0006351
PcpMADS57	GO:0005634,GO:0006355,GO:0046983,GO:0003677,GO:0006351
PcpMADS58	GO:0005634,GO:0006355,GO:0003700,GO:0016020,GO:0016021,GO:0046983,GO:0003677,GO:0006351
PcpMADS14	GO:0005634,GO:0006355,GO:0003700,GO:0016020,GO:0016021,GO:0046983,GO:0003677,GO:0006351
PcpMADS55	GO:0005634,GO:0006355,GO:0003700,GO:0009908,GO:0046983,GO:0030154,GO:0007275,GO:0003677,GO:0006351
PcpMADS11	GO:0005634,GO:0006355,GO:0003700,GO:0016020,GO:0016021,GO:0046983,GO:0009960,GO:0003677,GO:0006351
PcpMADS12	GO:0005634,GO:0006355,GO:0003700,GO:0046983,GO:0009960,GO:0003677,GO:0006351
PcpMADS56	GO:0005634,GO:0006355,GO:0046983,GO:0003677,GO:0006351
PcpMADS18	GO:0005634,GO:0006355,GO:0003700,GO:0016020,GO:0016021,GO:0046983,GO:0032440,GO:0055114,GO:0016491,GO:0003677,GO:0006351
PcpMADS59	GO:0010048,GO:0005634,GO:0010150,GO:0046983,GO:0030154,GO:0005515,GO:0007275,GO:0009838,GO:0006351,GO:0006355,GO:0003700,GO:0009908,GO:0009909,GO:0080187,GO:0003677,GO:0048510
PcpMADS15	GO:0005634,GO:0006355,GO:0003700,GO:0046983,GO:0032440,GO:0010154,GO:0055114,GO:0016491,GO:0003677,GO:0010077,GO:0006351
PcpMADS16	GO:0009793,GO:0048577,GO:0005634,GO:0060867,GO:0045892,GO:0045893,GO:0046983,GO:0005737,GO:0006351,GO:0006355,GO:0042803,GO:0010227,GO:0003700,GO:0009910,GO:0010262,GO:0003677,GO:0060862,GO:0010047
PcpMADS60	GO:0005634,GO:0000900,GO:0045892,GO:0046983,GO:0007275,GO:0006351,

	GO:0009266,GO:0006355,GO:0003700,GO:0009910,GO:0048438,GO:0017148, GO:0003677
PcpMADS61	GO:0005634,GO:0006355,GO:0046983,GO:0003677,GO:0006351
PcpMADS64	GO:0005634,GO:0006355,GO:0046983,GO:0003677,GO:0006351
PcpMADS20	GO:0005634,GO:0006355,GO:0046983,GO:0003677,GO:0006351
PcpMADS21	GO:0005634,GO:0006355,GO:0003700,GO:0046983,GO:0003677,GO:0006351
PcpMADS65	GO:0005634,GO:0006355,GO:0046983,GO:0003677,GO:0006351
PcpMADS62	GO:0005634,GO:0006355,GO:0003700,GO:0046983,GO:0010152,GO:0032440, GO:0055114,GO:0005515,GO:0016491,GO:0003677,GO:0080092,GO:0006351
PcpMADS63	GO:0005634,GO:0006355,GO:0016020,GO:0016021,GO:0046983,GO:0003677, GO:0006351
PcpMADS68	GO:0005634,GO:0006355,GO:0003700,GO:0016020,GO:0016021,GO:0046983, GO:0005515,GO:0003677,GO:0006351
PcpMADS24	GO:0005634,GO:0006355,GO:0046983,GO:0003677,GO:0006351
PcpMADS69	GO:0005634,GO:0006355,GO:0003700,GO:0046983,GO:0003677,GO:0006351
PcpMADS25	GO:0005634,GO:0008380
PcpMADS73	GO:0005634,GO:0008380
PcpMADS22	GO:0005634,GO:0006355,GO:0003700,GO:0046983,GO:0003677,GO:0006351
PcpMADS66	GO:0005634,GO:0006355,GO:0046983,GO:0003677,GO:0006351
PcpMADS23	GO:0005634,GO:0006355,GO:0003700,GO:0046983,GO:0003677,GO:0006351
PcpMADS67	GO:0048577,GO:0009793,GO:0005634,GO:0060867,GO:0045892,GO:0045893, GO:0046983,GO:0005737,GO:0006351,GO:0006355,GO:0009555,GO:0042803, GO:0010227,GO:0003700,GO:0009910,GO:0010262,GO:0003677,GO:0060862, GO:0010047
PcpMADS28	GO:0005634,GO:0006355,GO:0046983,GO:0003677,GO:0006351
PcpMADS29	GO:0005634,GO:0006355,GO:0046983,GO:0003677,GO:0006351
PcpMADS26	GO:0005634,GO:0006355,GO:0000166,GO:0003677,GO:0006351
PcpMADS27	GO:0005739
PcpMADS71	GO:0005634,GO:0006355,GO:0046983,GO:0003677,GO:0006351
PcpMADS72	GO:0005634,GO:0006355,GO:0046983,GO:0003677,GO:0006351
PcpMADS70	GO:0005634,GO:0006355,GO:0009555,GO:0016020,GO:250016021,GO:00469 83,GO:0032440,GO:0055114,GO:0016491,GO:0003677,GO:0006351
PcpMADS31	GO:0005634,GO:0006355,GO:0003700,GO:0046983,GO:0003677,GO:0006351
PcpMADS32	GO:0005634,GO:0006355,GO:0003700,GO:0046983,GO:0032440,GO:0055114, GO:0016491,GO:0003677,GO:0006351
PcpMADS30	GO:0005634,GO:0006355,GO:0003700,GO:0046983,GO:0003677,GO:0006351
PcpMADS17	

Table-S9: Putative functions of pear MADS-box transcription factors.

Subgroup	P. bretschneideri	P. communis	A .thaliana and other plants	Functions
Mα	PbrMADS03	PcpMADS04	AtAGL40	The development of female gametophyte and embryo[1-4]
	PbrMADS09	PcpMADS08	AtAGL62	
	PbrMADS11	PcpMADS09	AtAGL28	
	PbrMADS31	PcpMADS10	AtAGL23	
	PbrMADS34	PcpMADS11	AtAGL61	
	PbrMADS35	PcpMADS12	AtAGL91	
	PbrMADS38	PcpMADS27	AtAGL29	
	PbrMADS39	PcpMADS28	AtAGL57	
	PbrMADS64	PcpMADS29	AtAGL59	
	PbrMADS65	PcpMADS34	AtAGL88	
	PbrMADS67	PcpMADS35	AtAGL85	
	PbrMADS74	PcpMADS37	AtAGL58	
		PcpMADS38	AtAGL74	
		PcpMADS40	AtAGL39	
		PcpMADS42	AtAGL99	
		PcpMADS56	AtAGL55	
		PcpMADS61	AtAGL56	
		PcpMADS63	AtAGL97	
		PcpMADS67	AtAGL60	
			AtAGL83	
			AtAGL84	
			AtAGL73	
			AtAGL100	
Mβ	PbrMADS05	PcpMADS03	AtAGL103	Unknown
	PbrMADS25	PcpMADS13	AtAGL53	
	PbrMADS28	PcpMADS20	AtAGL93	
	PbrMADS62	PcpMADS52	AtAGL89	
	PbrMADS66	PcpMADS57	AtAGL54	
		PcpMADS71	AtAGL26	
			AtAGL101	
			AtAGL52	
			AtAGL78	
			AtAGL51	
			AtAGL43	
			AtAGL75	
			AtAGL76	

			AtAGL77	
			AtAGL105	
			AtAGL81	
			AtAGL98	
Mγ	PbrMADS10	PcpMADS05	AtAGL45	Endosperm development[5]
	PbrMADS17	PcpMADS24	AtAGL46	
	PbrMADS18	PcpMADS41	AtAGL80	
	PbrMADS19	PcpMADS46	AtAGL86	
	PbrMADS23	PcpMADS64	AtAGL92	
	PbrMADS27	PcpMADS65	AtAGL36	
	PbrMADS37	PcpMADS66	AtAGL90	
	PbrMADS40	PcpMADS72	AtAGL38	
	PbrMADS41		AtAGL41	
	PbrMADS42		AtAGL48	
	PbrMADS48		AtAGL95	
	PbrMADS59		AtAGL96	
	PbrMADS60		AtPHE1	
	PbrMADS61			
	PbrMADS68			
	PbrMADS72			
	PbrMADS73			
A	PbrMADS44	PcpMADS15	AtAP1	The occurrence and development of sepal and petal[6]
	PbrMADS51	PcpMADS43	AtAGL79	
		PcpMADS53		
	PbrMADS63	PcpMADS55	AtFUL	
		PcpMADS69	AtCAL	
B	PbrMADS07	PcpMADS06	AtAP3	Specifies petal and stamen identities[7]
	PbrMADS29	PcpMADS17	AtPI	
	PbrMADS53	PcpMADS19		
		PcpMADS50		
C/D	PbrMADS12	PcpMADS14	AtAG	Specifies stamen and carpel identities[8-10]
	PbrMADS13	PcpMADS26	AtSTK	
	PbrMADS32	PcpMADS30	AtSHP1	
	PbrMADS36	PcpMADS39	AtSHP2	
	PbrMADS50	PcpMADS58		
	PbrMADS56	PcpMADS68		

E	PbrMADS15	PcpMADS31	MdMADS8(MdMADS1)	Specifies flower meristem and organ identities,[11-15] anthocyanin biosynthesis[16-17]
	PbrMADS20	PcpMADS44	MdMADS9	
	PbrMADS45	PcpMADS47	AtSEP3	
	PbrMADS46	PcpMADS54	AtSEP4	
	PbrMADS58			
AGL12	PbrMADS26	PcpMADS25	AtAGL12	Root meristem cell proliferation and flowering transition[18]
	PbrMADS57	PcpMADS51		
AGL13	PbrMADS04	PcpMADS01	AtAGL6	Regulate floral organ identity and meristem fate[19]
			AtAGL13	
SOC1	PbrMADS01	PcpMADS02	AtSOC1	Floral promoter[20-21]
	PbrMADS02	PcpMADS59	AtAGL14	
	PbrMADS16		AtAGL19	
	PbrMADS22		AtAGL42	
	PbrMADS69		AtAGL71	
	PbrMADS70		AtAGL72	
	PbrMADS71			
SVP	PbrMADS24	PcpMADS21	AtSVP	Floral repressor[22-23]
	PbrMADS47	PcpMADS22	AtAGL24	Floral promoter[24]
	PbrMADS54	PcpMADS23		
		PcpMADS60		
Bs	PbrMADS33	PcpMADS07	AtTT16	Endothelium and seed color development[25-28]
		PcpMADS32	AtAGL63	
		PcpMADS33		
		PcpMADS36		
FLC	Loss	Loss	AtFLC	Floral repressor (vernalization)[29-31]
			AtMAF1	
			AtMAF2	
			AtMAF3	
			AtMAF4	
			AtMAF5	
ANR1	Loss	Loss	AtANR1	The repression of plant flowering time[32-33]
			AtAGL16	
			AtAGL17	

			AtAGL21	
AGL15/18	PbrMADS06	PcpMADS16	AtAGL15	Floral transition repressor[34]
	PbrMADS08	PcpMADS49	AtAGL18	
	PbrMADS21			
	PbrMADS49			
MIKC*	PbrMADS14	PcpMADS18	AtAGL30	Involved in late stages of pollen development and pollen tube growth[35-36]
	PbrMADS30	PcpMADS45	AtAGL33	
	PbrMADS43	PcpMADS48	AtAGL65	
	PbrMADS52	PcpMADS62	AtAGL66	
	PbrMADS55	PcpMADS70	AtAGL67	
			AtAGL94	
			AtAGL104	

References

1 Colombo, M. *et al.* AGL23, a type I MADS - box gene that controls female gametophyte and embryo development in Arabidopsis. *Plant Journal* **54**, 1037-1048 (2008).

2 Steffen, J. G., Kang, I. H., Portereiko, M. F., Lloyd, A. & Drews, G. N. AGL61 interacts with AGL80 and is required for central cell development in Arabidopsis. *Plant Physiology* **148**, 259-268 (2008).

3 Kang, I. H., Steffen, J. G., Portereiko, M. F., Lloyd, A. & Drews, G. N. The AGL62 MADS Domain Protein Regulates Cellularization during Endosperm Development in Arabidopsis. *Plant Cell* **20**, 635-647 (2008).

4 Portereiko, M. F. *et al.* AGL80 is required for central cell and endosperm development in Arabidopsis. *Plant Cell* **18**, 1862-1872 (2006).

5 Köhler, C. *et al.* The Polycomb-group protein MEDEA regulates seed development by controlling expression of the MADS-box gene PHERES1. *Genes Dev* **17**, 1540-1553 (2003).

6 Litt, A. An evaluation of A-function: Evidence from the APETALA1 and APETALA2 gene lineages. *International Journal of Plant Sciences* **168**, 73-91 (2007).

7 Whipple, C. J. *et al.* Conservation of B-class floral homeotic gene function between maize and Arabidopsis. *Development* **131**, 6083-6091 (2004).

8 Pnueli, L., Hareven, D., Rounsley, S. D., Yanofsky, M. F. & Lifschitz, E. Isolation of the tomato AGAMOUS gene TAG1 and analysis of its homeotic role in transgenic plants. *Plant Cell* **6**, 163 (1994).

9 Ray, A. *et al.* Arabidopsis floral homeotic gene BELL (BEL1) controls ovule development through negative regulation of AGAMOUS gene (AG). *Proceedings of the National Academy of Sciences of the United States of America* **91**, 5761-5765 (1994).

10 Favaro, R. *et al.* MADS-Box Protein Complexes Control Carpel and Ovule Development in Arabidopsis. *Plant Cell* **15**, 2603-2611 (2003).

11 Robles, P. The SEP4 gene of Arabidopsis thaliana functions in floral organ and meristem identity. *Current Biology* **14**, 1935-1940 (2004).

12 Ferrario, S., Immink, R. G., Shchennikova, A., Busscherlange, J. & Angenent, G. C. The MADS box gene FBP2 is required for SEPALLATA function in petunia. *Plant Cell* **15**, 914 (2003).

13 Pelaz, S., Ditta, G. S., Baumann, E., Wisman, E. & Yanofsky, M. F. B and C floral organ identity functions require SEPALLATA MADS-box genes. *Nature* **405**, 200-203 (2000).

14 Malcomber, S. T. & Kellogg, E. A. SEPALLATA gene diversification: brave new whorls. *Trends in Plant Science* **10**, 427-435 (2005).

15 Castillejo, C., Romerabranchat, M. & Pelaz, S. A new role of the Arabidopsis SEPALLATA3 gene revealed by its constitutive expression. *Plant Journal* **43**, 586-596 (2005).

16 Tapialópez, R. *et al.* An AGAMOUS-Related MADS-Box Gene, XAL1 (AGL12), Regulates Root Meristem Cell Proliferation and Flowering Transition in Arabidopsis. *Plant Physiology* **146**, 1182-1192 (2008).

17 Ohmori, S. *et al.* MOSAIC FLORAL ORGANS1, an AGL6-Like MADS Box Gene, Regulates Floral Organ Identity and Meristem Fate in Rice. *Plant Cell* **21**, 3008-3025 (2009).

18 Lee, J. & Lee, I. Regulation and function of SOC1, a flowering pathway integrator. *Journal of Experimental Botany* **61**, 2247-2254 (2010).

19 Moon, J. *et al.* The SOC1 MADS-box gene integrates vernalization and gibberellin signals for flowering in Arabidopsis. *Plant Journal* **35**, 613-623 (2003).

20 Lee, J. H. *et al.* Role of SVP in the control of flowering time by ambient temperature in Arabidopsis. *Genes Dev* **21**, 397-402 (2007).

21 Zachgo, S., Saedler, H. & Schwarz-Sommer, Z. Pollen‐specific expression of DEFH125, a MADS‐box transcription factor in Antirrhinum with unusual features. *Plant Journal* **11**, 1043-1050 (1997).

22 Liu, C. *et al.* Direct interaction of AGL24 and SOC1 integrates flowering signals in Arabidopsis. *Development* **135**, 1481-1491 (2008).

23 De Folter, S. *et al.* A B sister MADS-box gene involved in ovule and seed development in petunia and Arabidopsis. *Plant Journal* **47**, 934-946 (2006).

24 Becker, A. *et al.* A novel MADS-box gene subfamily with a sister-group relationship to class B floral homeotic genes. *Molecular Genetics & Genomics* **266**, 942-950 (2002).

25 Prasad, K., Zhang, X., Tobón, E. & Ambrose, B. A. The Arabidopsis B-sister MADS-box protein, GORDITA, represses fruit growth and contributes to integument development. *Plant Signaling & Behavior* **62**, 203-214 (2010).

26 Erdmann, R., Gramzow, L., Melzer, R., Theissen, G. & Becker, A. GORDITA (AGL63) is a young paralog of the Arabidopsis thaliana Bsister MADS box gene ABS (TT16) that has undergone neofunctionalization. *Plant Journal* **63**, 914–924 (2010).

27 Michaels, S. D. & Amasino, R. M. FLOWERING LOCUS C encodes a novel MADS domain protein that acts as a repressor of flowering. *Plant Cell* **11**, 949-956 (1999).

28 Michaels, S. D., He, Y., Scortecci, K. C. & Amasino, R. M. Attenuation of FLOWERING LOCUS C activity as a mechanism for the evolution of summer-annual flowering behavior in Arabidopsis. *Proceedings of the National Academy of Sciences of the United States of America* **100**, 10102-10107 (2003).

29 Michaels, S. D. & Amasino, R. M. Loss of FLOWERING LOCUS C Activity Eliminates the Late-Flowering Phenotype of FRIGIDA and Autonomous Pathway Mutations but Not Responsiveness to Vernalization. *The Plant Cell* **13**, 935-941 (2001).

30 Hu, J. Y. *et al.* miR824-Regulated AGAMOUS-LIKE16 Contributes to Flowering Time Repression in Arabidopsis. *Plant Cell* **26**, 2024 (2014).

31 Heuer, S., Lörz, H. & Dresselhaus, T. The MADS box gene ZmMADS 2 is specifically expressed

in maize pollen and during maize pollen tube growth. *Sexual Plant Reproduction* **13**, 21-27 (2000).

32 Adamczyk, B. J., Lehti-Shiu, M. D. & Fernandez, D. E. The MADS domain factors AGL15 and AGL18 act redundantly as repressors of the floral transition in Arabidopsis. *Plant Journal* **50**, 1007-1019 (2007).

33 Adamczyk, B. J. & Fernandez, D. E. MIKC* MADS domain heterodimers are required for pollen maturation and tube growth in Arabidopsis. *Plant Physiology* **149**, 1713-1723 (2009).

34 Verelst, W. & Münster, T. MIKC* MADS-protein complexes bind motifs enriched in the proximal region of late pollen-specific Arabidopsis promoters. *Plant Physiology* **143**, 447-460 (2007).

35 Feng, X., An, Y., Zheng, J., Sun, M. & Wang, L. Proteomics and SSH Analyses of ALA-Promoted Fruit Coloration and Evidence for the Involvement of a MADS-Box Gene, MdMADS1. *Frontiers in Plant Science* **7** (2016).

36 Ireland, H. S. *et al.* Apple SEPALLATA1/2-like genes control fruit flesh development and ripening. *Plant Journal* **73**, 1044–1056 (2013).

Table S10: The primer sequences used for qRT-PCR.

Gene Name	Forward primer	Reverse primer
PcpMADS02	TGAGAGGGAAGATTGAGATGAA	GTTAGAGTCTCTTGCATGTCAG
PcpMADS36	AAACGGCGGGCAGGACTGAT	AGCTCCTCCACGCTTGATGC
PcpMADS73	GTGGAACCTGCTCAGACGAAGT	CGATTTCAGCACGGTGTTTGGA
PcpMADS14	GCATCCGTTCTAAATCGAGACA	CTGGTGTTGCTGCGAGAAC
PcpMADS58	GGAGCTGAAGAGAATCGAGAAC	AATTGGTAGGACTTGCCAGATG
PcpMADS30	CCAAATCAAGCACCTGAGAG	GGAGAAGACAATAAGAGCAACT
PcpMADS26	CGAATCGGCAAGTGACCT	CACGAGTAGAGAAGACAATGAG
PcpMADS68	ATCCTTGTCAATCGACTCTC	CGGTTAGAGAAGACTATGAGA
PcpMADS59	GGAAGACGCAGATGAAGC	GTGTGTTGTTGATACTAGAGC
PcpMADS43	CTCAGCCCGAGGTTCAAA	GGTTCCAGAGTAAGGTCCAG
PcpMADS15	AGAGGATTGAGAACAAGGTC	GCAGGAATCAGTGGAGTAC
PcpMADS69	GCTGAAGCGAATCGAGAAC	GAGAAGACAATAAGTGCCACAT
PcpMADS01	GAGAGGGAGAGTGGTTATGGAG	ACGGCTGGAGTAGATGATAAGG
PcpMADS44	AGGTGGAGTTGAAGAGGAT	CGTCTTCGCTATGCTAGG
PcpMADS47	TGGGCAGAGGCAGAGTTGAG	CAGCATCGCAGAGAACTGAGAG
PcpMADS31	GCTGAAGAGGATAGAGAACAAG	AATGCTAGGGCTGCTACAAA
PcpMADS50	GGGTCGTGGAAAGATTGAGAT	CTAGTGTTGGAGAGCATGATGA
PcpMADS06	TGGGTCGTGGGAAGATTGAA	ACTCTTGGTCGTAGTGGTAGG
PcpMADS19	AACTCAAGTAACAGGCAGGT	CTGTCAGCGTAGTTGAAGG
PcpMADS60	CTGTGATGCCGATATTGCTCTT	TCCTTGCTCAACATGGTGTAGT
PcpMADS16	TAGCAGACAAGTCACATTCTC	AAGAGTTCGCATCATACCAG
PcpMADS18	GAATACGACCAATAGGCAAGT	GACTAAGTCTCCCAGATGAAG
PcpMADS62	GGTCGTGTTAAGCTGGAGATAA	CAATGTCAATGTCGCAAAGGAT

PcpMADS45	ACAAATGGGCGTCAAGCA	CGTCTTCAATACTACTGCGTTT
PcpMADS48	GGTGGACTATCAACTAAGTAGC	CTTGCCGAAACAGAACGA
PcpMADS70	TGAACATACCAGCAAATCCAG	CGATGGAAGGAATGCTCAC
PcpMADS34	ACTCTCTGTGGTGCTGAG	GTGAAGGTGGTGATTAGATGG
PcpMADS35	CGTCGTTCAGGGCTCTTCA	AAAGGAGAAGGGCTTGTTTGC
PcpMADS08	TACGAATGAGGCTAGTCTCCAA	AAGCACGGGTGACCAAAGG
PcpMADS38	ATGGAGGGCAAGCAAACAAG	AAGGCTTACCAGAAGGTGAGAA
PcpMADS04	ACTGGAGTCGGAAGAAGGAG	CAGGAGACAGCACTAGAACAG
PcpMADS03	TTTGGGAAAGAGGAGAGAC	CCAGTAATTGAGATGTTCGT
PcpMADS20	GGTGACTCCTACGAGAAGAAG	AGACAATCAAGGCCACATCAAT
PcpMADS72	TGTCCCGAAAGAAGGTGAAGC	ACAGCACATGCCTCAACATCA
PcpMADS66	AAAGGAAGAAGGGACTACTGAA	GCCAAACCTCAGCTTGAGTATC
PcpMADS24	CGTTCAAGAAGAGAAGGAAGG	GGAGTCATACGGGCTGTAAA
PcpMADS21	GACGGCGATCAAGGAGTT	CAAGATGTCGGAGCAGTCA
PbrMADS69	CAGAAGTTGCAGTCATCATC	GCACCTGTTGTTCCACTT
PbrMADS33	AAACGGCGGGCAGGACTGAT	AGCTCCTCCACGCTTGATGC
PbrMADS26	GTTGAGGAACTCGGAAGGA	GGTTAGTGGATATGTGTTGTCA
PbrMADS13	TGAACGCATCCGCTCTAA	CGAGAACATAACCTTGGAATG
PbrMADS50	CCAACAAGCAGACAGGTCAC	GGTAGGACTTGCCAGATGGA
PbrMADS32	CCAAATCAAGCACCTGAGAG	GGAGAAGACAATAAGAGCAACT
PbrMADS56	TCCATTCTCTGTGATGCTGAA	TGCCGATTCCTGTTGATAATAC
PbrMADS12	TCCTTGTCAATCGACTCTCC	GCATCACAGAGCACAGAGA
PbrMADS16	GGAAGACGCAGATGAAGC	GTGTGTTGTTGATACTAGAGC
PbrMADS44	GGCTGCTGAAGAAGGCTCAT	GCATGAATCTGTGGCGTACTC
PbrMADS51	TGAAGAGGATTGAGAACAAG	GCAGGAATCATTGGAGTAC
PbrMADS63	GCTGAAGCGAATCGAGAAC	GAAGACAATAAGTGCCACATCA
PbrMADS04	AGAACAAGATTAACAGGCAGGT	CCACGGCTAGAGAAGATGATAA
PbrMADS45	GGAAGGTGGAGTTGAAGAGGAT	CTGGTGGAGAAGACGATGAGAG
PbrMADS15	GTTGAGTTGAAGAGGATAGAG	CATGCTTAAACTGCTTGAGA
PbrMADS20	AGGTTGCTCTCATCATCTTCTC	ACGATAGCTGCTCTCAAGTTC
PbrMADS53	GGGTCGTGGAAAGATTGAGAT	CTAGTGTTGGAGAGCATGATGA
PbrMADS07	GGGTCGTGGGAAGATTGA	AGTGTTGGAGAGCATAATGAG
PbrMADS29	CTCAAGTAACAGGCAGGTG	CTGTCAGCGTAGTTGAAGG
PbrMADS47	CTGTGATGCCGATATTGCTCTT	TCCTTGCTCAACATGGTGTAGT
PbrMADS21	TAGCAGACAAGTCACATTCTC	AAGAGTTCGCATCATACCAG
PbrMADS30	GAATACGACCAATAGGCAAGT	GACTAAGTCTCCCAGATGAAG
PbrMADS55	GGTCGTGTTAAGCTGGAGATAA	CAATGTCAATGTCGCAAAGGAT
PbrMADS43	AATACAAATGGGCGTCAAGCA	CGTCTTCAATACTGCGTTTGC
PbrMADS52	ACAAGGCGATGAGCGATGG	AGTTGAAGTGCAGTACAAGGAG
PbrMADS14	AGAAACTAGAGAGCAGTGG	CCCTTGGAATAATGTAGGC

PbrMADS39	TCTCTGTGGTGCTGAGGT	GGAGAGGAATCGGTCTATGA
PbrMADS38	AACGTCGTTCAGGGCTCTT	CCAAAGGAGAAGGGCTTGTTT
PbrMADS11	TTACGAATGAGGCTAGTCTCC	CACGGGTGACCAAAGGAA
PbrMADS35	GGGCAAGCAAACAAGAGGAA	AAGGCTTACCAGAAGGTGAGAA
PbrMADS09	AACTGGAGTCGCAAGAAGGAG	GCAGGAGACAGCACTAGAACG
PbrMADS05	GCGGGAGGCTGAATATGGAA	CGTGTGGAGTTCGGGACTAC
PbrMADS25	TGGTGACTCCTACGAGAAGAAG	ATGTCTCCGGCTGAACTGG
PbrMADS17	GAAGATGAGCGAAATCACTACC	CTTTGTACTCCCGAAGGAAATG
PbrMADS19	AAAGGAAGAAGGGACTACTGAA	GCCAAACCTCAGCTTGAGTATC
PbrMADS23	CGTTCAAGAAGAGAAGGAAGG	GGGAGTCATAAGGGCTGTAAA
PbrMADS24	CTGAAGAGCTGTCGATTCTGT	CTTACTGAGCCTGATCGTGTT
